# 6-Bromoindole Derivatives from the Icelandic Marine Sponge *Geodia barretti*: Isolation and Anti-Inflammatory Activity

**DOI:** 10.3390/md16110437

**Published:** 2018-11-08

**Authors:** Xiaxia Di, Caroline Rouger, Ingibjorg Hardardottir, Jona Freysdottir, Tadeusz F. Molinski, Deniz Tasdemir, Sesselja Omarsdottir

**Affiliations:** 1 Faculty of Pharmaceutical Sciences, University of Iceland, Hagi, Hofsvallagata 53, IS-107 Reykjavik, Iceland; xid1@hi.is; 2 Department of Immunology and Centre for Rheumatology Research Landspitali-The National University Hospital of Iceland, Hringbraut, IS-101 Reykjavik, Iceland; ih@hi.is (I.H.); jonaf@landspitali.is (J.F.); 3 GEOMAR Centre for Marine Biotechnology (GEOMAR-Biotech), Marine Natural Products Chemistry Research Unit, GEOMAR Helmholtz Centre for Ocean Research Kiel, Am Kiel-Kanal 44, 24106 Kiel, Germany; caroline.rouger@u-bordeaux.fr (C.R.); dtasdemir@geomar.de (D.T.); 4 Faculty of Medicine, Biomedical Center, University of Iceland, Vatnsmyrarvegur 16, IS-101 Reykjavik, Iceland; 5 Department of Chemistry and Biochemistry and Skaggs School of Pharmacy and Pharmaceutical Sciences, University of California, San Diego, La Jolla, CA 92093, USA; tmolinski@ucsd.edu; 6 Faculty of Mathematics and Natural Sciences, Kiel University, Christian-Albrechts-Platz 4, 24118 Kiel, Germany

**Keywords:** 6-bromoindole, *Geodia barretti*, anti-inflammatory activity, dendritic cells, T cell differentiation

## Abstract

An UPLC-qTOF-MS-based dereplication study led to the targeted isolation of seven bromoindole alkaloids from the sub-Arctic sponge *Geodia barretti*. This includes three new metabolites, namely geobarrettin A–C (**1**–**3**) and four known compounds, barettin (**4**), 8,9-dihydrobarettin (**5**), 6-bromoconicamin (**6**), and l-6-bromohypaphorine (**7**)*.* The chemical structures of compounds **1**–**7** were elucidated by extensive analysis of the NMR and HRESIMS data. The absolute stereochemistry of geobarrettin A (**1**) was assigned by ECD analysis and Marfey’s method employing the new reagent l-*N*^α^-(1-fluoro-2,4-dinitrophenyl)tryptophanamide (l-FDTA). The isolated compounds were screened for anti-inflammatory activity using human dendritic cells (DCs). Both **2** and **3** reduced DC secretion of IL-12p40, but **3** concomitantly increased IL-10 production. Maturing DCs treated with **2** or **3** before co-culturing with allogeneic CD4^+^ T cells decreased T cell secretion of IFN-γ, indicating a reduction in Th1 differentiation. Although barettin (**4**) reduced DC secretion of IL-12p40 and IL-10 (IC_50_ values 11.8 and 21.0 μM for IL-10 and IL-12p40, respectively), maturing DCs in the presence of **4** did not affect the ability of T cells to secrete IFN-γ or IL-17, but reduced their secretion of IL-10. These results indicate that **2** and **3** may be useful for the treatment of inflammation, mainly of the Th1 type.

## 1. Introduction

Many chronic illnesses, including cancer, neurological diseases, diabetes, and autoimmune diseases, exhibit dysregulation of pathways that have been linked to inflammation [1,2]. A vast number of unique marine natural products possessing in vitro and in vivo anti-inflammatory activity have been isolated [3,4], however, no marine-derived anti-inflammatory agent is currently on the market. Therefore, the search and development of new natural products to treat chronic inflammatory diseases is of great importance [5,6]. Marine sponges, due to their phenomenal biological and chemical diversity, are considered as a notable source of natural products with potential anti-inflammatory activity [5,7,8].

*Geodia barretti* Bowerbank (family Geodiidae, class Demospongiae, order Astrophorida) is an abundant cold-water sponge with a wide geographic distribution, including the Icelandic waters. *G. barretti* has been reported to contain 6-bromoindole alkaloids [9,10,11,12,13], including barettin (**4**) [10], 8,9-dihydrobarettin (**5**) [11], bromobenzisoxazolone barettin [12], 6-bromoconicamin (**6**) [14], and 2-(6-bromo-1*H*-indol-3-yl)-2-hydroxy-*N*,*N*,*N*-trimethylethanaminium [13], as well as nucleosides [15], peptides [16], sterols and fatty acids [17].

The chemical structure of barettins isolated from *G. barretti* feature a diketopiperazine (DKP)-type cyclic dipeptide, a condensation product of arginine (Arg) and 6-bromo-8-en-tryptophan residues in a “head-to-tail” fashion [11,18]. The DKP core, present in many bioactive compounds, is a relevant scaffold for drug discovery and development [19]. Barettins display interesting biological activities, such as anti-inflammatory, antioxidant, antifouling, acetylcholinesterase (AChE) inhibitory, and selective serotonin (5-HT) receptor ligand effects [11,12,13,20,21]. In particular, barettin (**4**), the major compound in *G. barretti* has shown potent antifouling, antioxidant, and anti-inflammatory activities [11,12,20], making it a potential lead compound in prevention of chronic inflammatory diseases. Therefore, the search for new barettins is of great interest for investigation of their anti-inflammatory effects and structure–activity relationships. 

The present study was undertaken to isolate 6-bromoindole derivatives from the Icelandic sponge *G. barretti* and to evaluate their anti-inflammatory activity on human monocyte-derived dendritic cells (DCs) and their ability to activate T cell responses. DCs play an important role in initiation of adaptive immune responses by activating naïve T cells and inducing their differentiation by cytokine secretion [22]. The naïve CD4^+^ T cells differentiate into different T helper (Th) effector cells, such as Th1, Th2, Th9, or Th17 phenotypes, which participate in various inflammatory diseases [23,24,25] or, if the DCs secrete anti-inflammatory cytokines, such as IL-10, into T regulatory cells (Tregs) [26]. In this study, we describe (i) the isolation and structure elucidation of three new 6-bromoindole derivatives, geobarrettin A–C (**1**–**3**) and four known 6-bromoindole alkaloids (**4**–**7**), (ii) the absolute stereoassignment of geobarrettin A (**1**) using the Marfey’s method employing the new reagent l-*N*^α^-(1-fluoro-2,4-dinitrophenyl)tryptophanamide (l-FDTA) followed by HPLC comparison with standards, and (iii) the re-evaluation of the stereochemical configuration of barettin (**4**). 

## 2. Results

**Figure 1:**
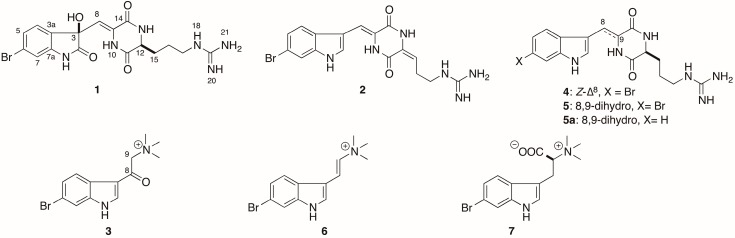
Chemical structures of compounds **1**–**7** and **5a**.

The sponge *G. barretti* was collected at the west of Iceland (−388 m) and kept frozen until work-up. The CH_2_Cl_2_:CH_3_OH (*v*/*v*, 1:1) soluble extract of the sponge was partitioned into five subextracts (hexane, chloroform, dichloromethane, *n*-butanol, and water) using a modified Kupchan solvent partitioning method [27,28]. The crude extracts and subextracts were chemically profiled by UPLC-qTOF-MS for selective and sensitive detection of bromoindoles and other trace components [29,30]. The bromoindole containing chloroform and dichloromethane subextracts were combined and purified by a combination of RP-VLC and HPLC to afford three new 6-bromoindole derivatives geobarrettin A–C (**1**–**3**) and four known alkaloids **4**–**7** (Figure 1).

### 2.1. Structural Elucidation

**Figure 2:**
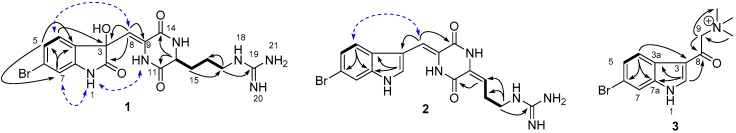
Key HMBC (H→C), ^1^H-^1^H COSY (−) and NOESY (H↔H, in dashed blue) correlations of compounds **1**–**3**.

Compound **1** was obtained as a yellow solid. Its UV spectrum was characteristic for an oxindole skeleton with absorption λ_max_ at 217 and 263 nm [31,32], while the IR spectrum showed the presence of OH (ν_max_ 3352 cm^−1^), NH_2_ (ν_max_ 3285, 3214, and 1614 cm^−1^), and lactam (ν_max_ 1679 cm^−1^) functionalities. The characteristic isotope pseudo-molecular ion peaks at *m*/*z* 451.0728:453.0732 ([M + H]^+^) in a 1:1 ratio in the HRESIMS spectrum of **1** suggested the presence of a bromine atom in the molecule. The HRESIMS data indicated the molecular formula of C_17_H_20_^79^BrN_6_O_4_, corresponding to eleven degrees of unsaturation. This formula was supported by the ^1^H, ^13^C NMR, and HSQC data, indicating the presence of three methylenes (δ_C_ 42.0, 32.6 and 24.8), three aromatic methines (δ_C_ 127.1, 126.9 and 115.0), one aliphatic methine (δ_C_ 56.0), an isolated olefinic methine (δ_C_ 112.8), four quaternary aromatic carbons (δ_C_ 143.9, 132.4, 132.2, and 124.7), one tertiary alcohol (δ_C_ 78.3), and four quaternary heteroatom-bounded sp^2^ carbons (δ_C_ 179.3, 167.2, 160.5 and 158.7). The ^1^H NMR spectrum in CD_3_OD contained three aromatic resonances, including a broad singlet at δ_H_ 7.11 (1H) and an overlapped 2H broad singlet at δ_H_ 7.25 (Table 1). When acquired in DMSO-*d*_6_, these aromatic signals were resolved into a well-separated ABX system at δ_H_ 7.26 (1H, d, *J* = 7.9 Hz, H-4), 7.23 (1H, dd, *J* = 7.9, 1.5 Hz, H-5), and 7.03 (1H, d, *J* = 1.5 Hz, H-7), suggesting the presence of a trisubstituted benzene ring. The chemical shift of the quaternary aromatic carbon C-7a (δ_C_ 143.9) indicated that C-7a was substituted by an NH group. The characteristic resonances of the amide carbonyl C-2 at δ_C_ 179.3 and the oxygenated carbon (C-3, δ_C_ 78.3), as well as the HMBC correlations observed from H-4 to C-3, C-6, and C-7a, and from H-7 to C-3a, assisted the construction of a 3-hydroxy-2-oxindole skeleton. The chemical shifts for carbon atoms were in good agreement with those of previously published 3-hydroxy-2-oxindole scaffolds [33,34]. The position of the bromine atom at C-6 was determined based on the HMBC correlations H-4/C-3, H-4/C-6, and H-4/C-7a; H-5/C-3a and H-5/C-7; as well as H-7/C-3a, H-7/C-5, and H-7/C-6. This was supported by the NOE correlations between H-4/H-8 observed in spectrum run in CD_3_OD and a weak cross peak between H-7/NH-1 in spectrum run in DMSO-*d*_6_, in combination with characteristic coupling constant values, namely *J*_4,5_ (7.9 Hz) and *J*_5,7_ (1.5 Hz). A similar weak cross peak between H-7/NH-1 has previously been observed for 6-bromoindole cyclic guanidine alkaloids [35]. The presence of two amide carbonyl signals C-11 (δ_C_ 167.2) and C-14 (δ_C_ 160.5) suggested the presence of a DKP moiety attached to the oxindole skeleton and the chemical shift of C-14 indicated that this carbon was conjugated to a double bond (Δ^8^). Cross correlations observed in the HMBC spectrum between H-12/C-11, H-12/C-14, H-8/C-14, H-8/C-3, and H-8/C-9 supported this assumption. ^1^H-^1^H COSY spectrum indicated the presence of an additional linear spin system starting from H-12 (δ_H_ 4.28, 1H, t, *J* = 6.0 Hz) and including three aliphatic methylene signals H_2_-15 (δ_H_ 1.89, 1H, m; δ_H_ 2.01, 1H, m), H_2_-16 (δ_H_ 1.67, 1H, m; 1.74, 1H, m), and H_2_-17 (δ_H_ 3.24, 2H, t, *J* = 6.0 Hz). The molecular fragments described above account for all atoms except three nitrogen atoms and one quaternary carbon atom (δ_C_ 158.7, C-19) and for 10 of 11 degrees of unsaturation. The position of the remaining three nitrogen atoms and the carbon atom was assigned to a guanidine group connected to the linear spin system at C-17 based on the HMBC correlation between H_2_-17/C-19 (Figure 2). This suggested that the aliphatic side chain linked to the DKP moiety was an Arg residue. Further examination of the NMR data revealed that spectroscopic data of compound **1** were very similar to those of compound **4** [10], with the main differences being the upfield shift of the H-7 resonance (δ_H_ 7.03 (1H d, *J* = 1.5 Hz) in compound **1**; δ_H_ 7.67 (1H d, *J* = 1.6 Hz) in compound **4**) and the replacement of the indole Δ^2^ double bond by a hydroxyl group at C-3 and a carbonyl group at C-2. The latter was confirmed by the HMBC correlation observed between H-8 and C-2 (Figure 2). The structure proposed for compound **1** is consistent with its molecular formula (*m*/*z* 451.0728 [M + H]^+^, C_17_H_20_^79^BrN_6_O_4_) in comparison to that of compound **4** (*m*/*z* 419.0830 [M + H]^+^, C_17_H_20_^79^BrN_6_O_2_). The geometry of the exocyclic double bond Δ^8^ was defined as *Z* based on NOESY correlations observed in H-8/H-4 and NH-10/NH-1.

**Figure 3:**
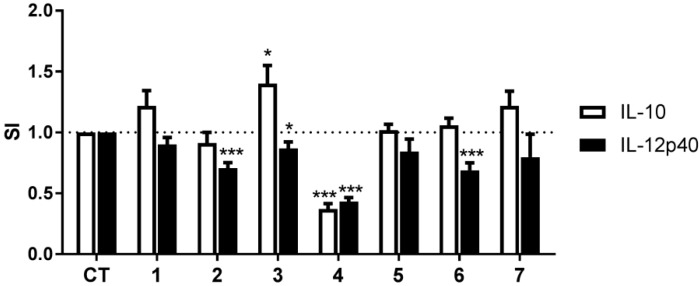
Comparison of CD of model compound (*R*)-3-propyldioxindole (**8**) and debromodihydrogeobarrettin A (**1a**). Red line: (*R*)-**8**; Blue line: **1a**.

**Scheme 1:**
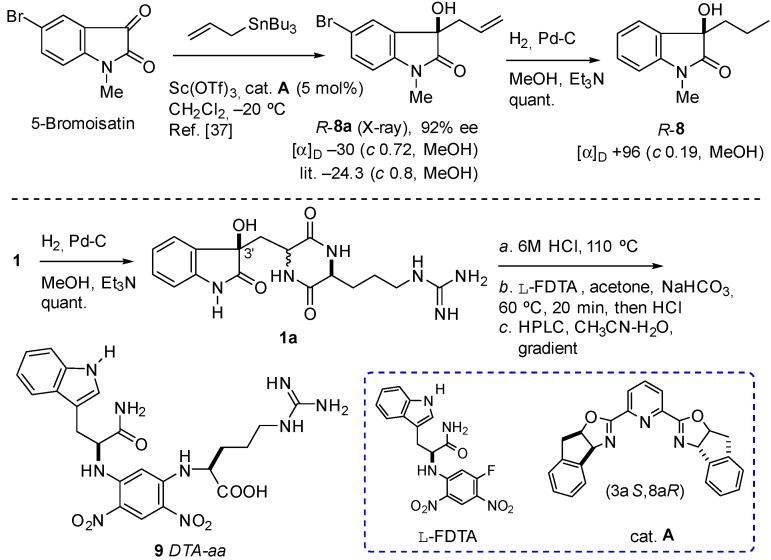
Hydrogenolysis of (*R*)-(**8a**) to (*R*)-(**8**) and hydrogenation, hydrolysis, and l-FDTA-derivatization of geobarrettin A (**1**) to **9**.

The configurations of two chiral centers at C-3 and C-12 in compound **1** were determined using a combination of techniques. The absolute configuration of C-3 was determined by comparison of the ECD spectra (Figure 3) of the hydrogenolysis product of compound **1** with the compound (*R*)-3-propyldioxindole (**8**), which share the dioxindole core structure. (*R*)-**8** was obtained by hydrogenolysis of *R*-dioxindole (**8a**, a gift from Dr. Annaliese K. Franz (University of California, Davis)) (Scheme 1) [37]. The CD spectra of (*R*)-**8** (EtOH, 23 °C) revealed Cotton effects (λ 208 nm (Δε +13.1), 232 (−10.7), 259 (+3.5)) similar to dioxindoles recently investigated [38]. Hydrogenolysis of geobarretin A (**1**) (Scheme 1) gave debromodihydrogeobarrettin A (**1a**) with a CD spectrum identical to that of (*R*)-**8** (Figure 3). Given the identical CD spectra of **1a** and (*R*)-**8**, whose absolute configuration was established by X-ray crystallography, and the similarity of their Cotton effects with those of (*R*)-5-methyldioxindole [39] and the natural product (+)-trikentramide I [38], the C-3 configuration of compound **1** was assigned as *R*, unambiguously.

Determination of amino acid configuration using Marfey’s method and chiral derivatizing agents (CDAs) such as l-fluoro-2-4-dinitrophenyl-5-l-alanine amide (FDAA, Marfey’s reagent) or the homolog, l-fluoro-2,4-dinitrophenyl-5-l-leucineamide (FDLA), are popular and effective [40]. Compound **1a** was hydrolyzed (6 M HCl, 110 °C) and the product was derivatized with the new CDA, l-FDTA [41], to produce **9**. Comparison with standards d- and l-Arg-DTA derivatives was then achieved using HPLC (Figure S32) and UPLC-MS analyses. d- and l-Arg-DTA derivatives were observed with integral values of 19:81 suggesting either natural **1** is partially racemic at C-12, or that Arg underwent partial racemization under the conditions of hydrolysis. The latter possibility is ruled out by precedence; Arg essentially is stable to the conditions of hydrolysis. In a careful systematic study Kaiser and Benner (2005) demonstrated that the amount of d-Arg produced during acid hydrolysis of l-Arg (110 °C, 6 M HCl, 20 h) is not more than 1.0 ± 0% of the total for free amino acid, or 2.8 ± 0.4% when bonded in lysozyme [42]. We conclude that compound **1** is a cryptic mixture of diastereomers with stereofidelity at C-3, but partial epimerization at C-12. Therefore, the configuration of geobarrettin A (**1**) is 3*R*, 8*Z*, 12*S*.

Geobarrettin B (**2**) was isolated as a yellow solid. The UV spectrum showed the characteristic indole chromophore with absorptions at λ_max_ 230, 270, and 289 nm [43]. Its molecular formula C_17_H_18_^79^BrN_6_O_2_ was deduced by HRESIMS (*m*/*z* 417.0675/419.0685 1:1, [M + H]^+^), indicating the presence of a bromine atom and 12 degrees of unsaturation. Detailed analysis of the ^1^H, ^13^C NMR (Table 1) and HSQC data suggested the planar structure of compound **2** to be very similar to that of the known compound barettin (**4**). Comparison of the 1D NMR spectra of compounds **2** and **4** suggested that the only difference was the presence of a double bond in the Arg side chain of **2**, namely between C-12 (δ_C_ 130.5) and C-15 (δ_C_ 114.9). The HMBC correlations from H-15 (δ_H_ 5.98, t, *J* = 7.8 Hz) to C-11 and C-17, and from H-16 (δ_H_ 2.57, q, *J* = 7.8 Hz) to C-12, as well as COSY correlations between H-15/H_2_-16 and H_2_-16/H_2_-17 supported the position of the unsaturation at Δ^12^. Finally the two mass units difference between the molecular formulae of **2** and **4**, as determined by HRESIMS data, clearly supported **2** being the ∆^12^ unsaturated analog of **4**. The geometry of Δ^12^ in α,β-dehydroamino-acids was determined by analyzing the reported trends in chemical shift differences of vinyl protons in *E*- and *Z*-isomers. Comparison of the chemical shift of H-15 (δ_H_ 5.98) in compound **2** with that of alkylidene-2,5-piperazinedione derivatives reported in the literature [44,45] therefore supports an *E*-Δ^12^ configuration. Hence compound **2** is the Δ^12^ dehydro-derivative of barettin (**4**), which we named as geobarrettin B.

Geobarrettin C (**3**) was obtained as a yellow oil. The molecular formula C_13_H_16_BrN_2_O^+^ was assigned to compound **3** by its HRESIMS data. Analysis of the ^1^H and ^13^C NMR spectra pointed out the presence of a substituted 6-bromoindole scaffold. The substituent was identified as *N*,*N*,*N*-trimethyl-2-oxoethanaminium salt, based on the 1D NMR spectra that possessed resonances of a methylene group at δ_C_ 67.8 and δ_H_ 4.92 (2H, s, H-9), a conjugated keto group at δ_C_ 186.0 (C-8), and three methyl signals at δ_C_ 54.9; δ_H_ 3.42 (9H, s, -N(CH_3_)_3_). The HMBC correlations observed between CH_3_-N/C-9, H_2_-9/C-8, and H-2/C-8 indicated the *N*,*N*,*N*-trimethyl-2-oxoethanaminium moiety to be attached to 6-bromoindole at C-3 position (δ_C_ 116.1). A literature search showed that **3** is an oxidation product of a previously isolated secondary alcohol 2-(6-bromo-1*H*-indol-3-yl)-2-hydroxy-*N*,*N*,*N*-trimethylethanaminium (C_13_H_18_BrN_2_O^+^) [13]. Thus, compound **3** was identified as the quaternary ammonium salt 2-(6-bromo-1*H*-indol-3-yl)-*N*,*N*,*N*-trimethyl-2-oxoethanaminium and named as geobarrettin C (**3**).

The gross structures of the remaining known compounds, barettin (**4**) [10], 8,9-dihydrobarettin (**5**) [11], 6-bromoconicamin (**6**) [14], and l-6-bromohypaphorine (**7**) [46] were identified by comparison of their NMR, HRMS, and specific rotation data with those reported in the literature.

The sole chiral amino acid residue in barettin (**4**), first reported in 1986 from a deep-water specimen of *G. barretti* collected in Sweden, presumably from Koster fjord [12], was assigned as Pro, but the configuration was not determined at that time. A second report of **4** in 2004 from a Norwegian deep-water sample of *G. barretti* [11] corrected the structure to an Arg-containing DKP, but also omitted the specific rotation. Finally, the configuration of **4** was established as l-Arg by comparison of optical rotations of natural and synthetic **4** [42]. Although they had similar signs, the specific rotations of synthetic and natural **4** were different (${\left[\alpha \right]}_{D}^{26}$ −32.5 (*c* 2, MeOH) [18]; natural **4** [α]_D_ −25 (*c* 3, MeOH)) [9]. In the present study, the rotation of **4** was found to be even higher (${\left[\alpha \right]}_{D}^{25}$ −84 (*c* 0.5, MeOH)). In order to resolve this paradox, the l-FDTA method was applied to the hydrolysate as described above for **1**. HPLC analysis detected the presence of d-Arg-DTA and l-Arg-DTA in a ratio of 19:81 suggesting l-Arg was predominant, but—as with **3**—our sample of **4** was also partially racemic. Assuming no racemization occurred during the synthesis of **4** [42], our finding would be consistent with partially racemic natural **4** in the Swedish sample. The hydrolysis was repeated on debromo-8,9-dihydrobarettin (**5a**) (Figure 1), obtained by hydrogenolysis of **4**, and the product was again converted to l-DTA derivatives and analyzed by HPLC as before to give d-Arg-DTA and l-Arg-DTA (19:81).

The structure of compound **7** was assigned as 6-bromohypaphorine by comparing the recorded and published NMR data [46]. The sign of optical rotation of compound **7** (${\left[\alpha \right]}_{D}^{23}$ +36, *c* 0.2, EtOH) was similar to that of l-6-bromohypaphorine ([α]_D_ +58, *c* 0.25, EtOH) [46] and opposite to that of the d-6-bromohypaphorine (${\left[\alpha \right]}_{D}^{17}$ −27, *c* 0.80, MeOH-CF_3_COOH, 8:1) [47]. Hence, compound **7** was assigned as l-6-bromohypaphorine.

### 2.2. Anti-Inflammatory Activity

**Figure 4:**
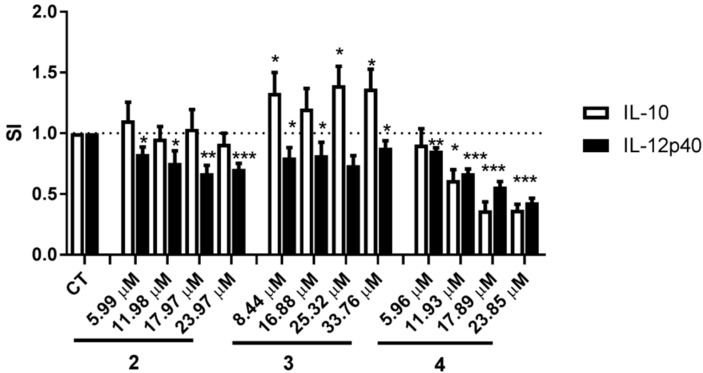
The effects of compounds **1**–**7** on dendritic cell (DC) secretion of IL-12p40 and IL-10. DCs were matured and activated by TNF-α, IL-1β and LPS in the absence (solvent control (CT)) or presence of compounds **1**–**7** at 10 μg/mL for 24 h. The supernatants were collected and the concentrations of IL-12p40 and IL-10 were determined by ELISA. The data are presented as SI, i.e., the concentration of each cytokine in the supernatant of cells matured and activated in the presence of compounds divided by the concentration of the cytokine in the supernatant of cells matured and activated in the presence of solvent control. The results are shown as mean + standard error of the mean (SEM), *n* = 7–11, except for compound **7**, *n* = 3. Different from CT: * *p* < 0.05, *** *p* < 0.001.

To evaluate the potential anti-inflammatory activity of compounds **1**–**7**, their effects on DC secretion of the pro-inflammatory cytokine IL-12p40 and the anti-inflammatory cytokine IL-10 was determined. The tested compounds did not show cytotoxic effects on the DCs, with the cell viability being more than 90% in the presence of the compounds at 10 μg/mL (data not shown). Compound **4** inhibited by more than 50% secretion of both IL-12p40 and IL-10. Compounds **2** and **6** decreased DC secretion of IL-12p40 by 29 and 32%, respectively, without affecting secretion of IL-10, which suggests an overall anti-inflammatory activity of these compounds. Compound **3** slightly decreased DC secretion of IL-12p40 (13%) but increased secretion of the anti-inflammatory cytokine IL-10 by 40%, which also suggests an anti-inflammatory activity. Compounds **1**, **5,** and **7** did not affect DC secretion of IL-12p40 or IL-10 (Figure 4).

**Figure 5:**
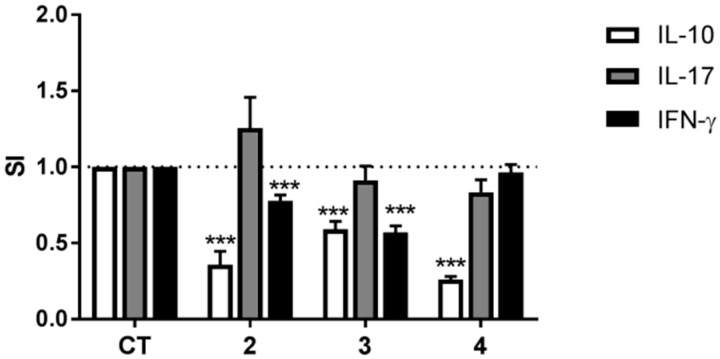
Dose-dependent inhibition of cytokines IL-12p40 and IL-10 by compounds **2**–**4**. DCs were matured and activated by TNF-α, IL-1β, and LPS in the absence (solvent control (CT)) or presence of indicated concentration of compound **2** (5.99, 11.98, 17.97, and 23.97 μM), compound **3** (8.44, 16.88, 25.32, and 33.76 μM), and compound **4** (5.96, 11.93, 17.89, and 23.85 μM). The levels of IL-12p40 and IL-10 in the supernatants were determined by ELISA. The data are presented as SI. The results are shown as mean + SEM, *n* = 5–11. Different from CT: * *p* ˂ 0.05, ** *p* ˂ 0.01, *** *p* ˂ 0.001.

We next examined whether compounds **2**, **3,** and **4** affected cytokine secretion by the DCs in a dose-dependent manner. The effects of compounds **2** and **3** on IL-12p40 and IL-10 secretion by DCs were not dose-dependent although for compound **2** there was a tendency towards an increasing effect on IL-12p40 secretion at higher concentrations. Compound **4** inhibited secretion of IL-12p40 and IL-10 in a dose-dependent manner with IC_50_ being 21.04 μM for IL-12p40 and 11.80 μM for IL-10 (Figure 5), with IL-12p40 and IL-10 secretion decreasing at the lowest concentrations tested, i.e., 5.96 μM (2.5 μg/mL) and 11.93 μM (5 μg/mL), respectively.

**Figure 6:**
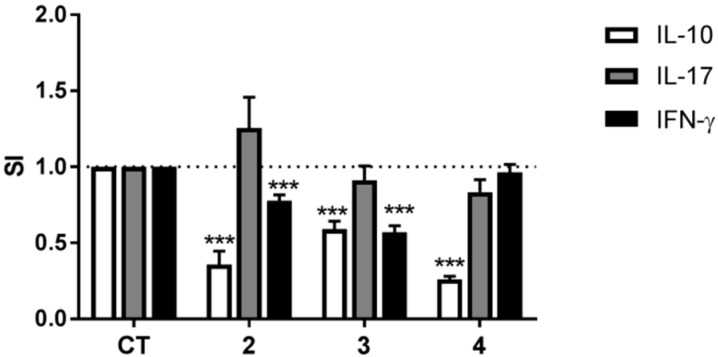
The effects of compounds **2**–**4** on the ability of DCs to induce cytokine secretion by allogeneic CD4^+^ T cells. DCs matured and activated in the absence (solvent control (CT)) or presence of compounds **2**–**4** at a concentration of 10 μg/mL for 24 h were co-cultured with isolated allogeneic CD4^+^ T cells for six days and the concentrations of IFN-γ, IL-17, and IL-10 in the supernatants were determined by ELISA. The data are presented as SI, i.e., the concentration of each cytokine in the supernatant of co-cultures containing DCs treated with compounds divided by the concentration of each cytokine in the supernatant of co-cultures containing DCs treated with solvent control. The results are shown as mean + SEM, *n* = 9–12. Different from CT: *** *p* < 0.001.

To further elucidate the anti-inflammatory activity of compounds **2**–**4**, DCs matured in their presence were co-cultured with allogeneic CD4^+^ T cells and the differentiation of the T cells investigated by determining secretion of the cytokines IL-10, IL-17, and IFN-γ. T cells co-cultured with DCs matured in the presence of compounds **2** or **3** secreted less IL-10 and IFN-γ than T cells co-cultured with DCs matured in the absence of the compounds, but maturing DCs in the presence of compounds **2** or **3** did not affect T cell secretion of IL-17 (Figure 6). Co-culturing T cells with DCs matured in the presence of compound **4** resulted in a substantial decrease in secretion of IL-10 with no effect on secretion of either IL-17 or IFN-γ.

## 3. Discussion

Three new 6-bromoindole derivatives were isolated from *G. barretti*, including a dioxindole featuring a DKP-type cyclic dipeptide, geobarrettin A (**1**); a 6-bromoindole possessing DKP system, geobarrettin B (**2**); and a new 6-bromoindole alkaloid, geobarrettin C (**3**).

Compounds **2** and **3** inhibited IL-12p40 production by DCs and DCs treated with compounds **2** and **3** reduced IFN-γ secretion by co-cultured T cells, hence reduced Th1 responses, which are linked to inflammatory disorders and many chronic inflammatory diseases [48,49]. As IL-12 is the main inducer of Th1 polarization of T cells with subsequent IFN-γ secretion [50], the down-regulation of IFN-γ observed in the co-culture of T cells with DCs is most likely resulting from a reduced ability of the DCs matured in the presence of compounds **2** and **3** to secrete IL-12p40 (one of the two chains that form the IL-12 molecule). Compound **2** did not affect IL-10 secretion by DCs but compound **3** increased IL-10 secretion by DCs. Therefore, the decreased concentration of IL-10 in co-cultures of T cells and DCs matured in the presence of compounds **2** and **3** was unexpected. The reduced IL-10 levels observed in the co-cultures were most likely the result of reduced secretion by the T cells but not the DCs, but whether it is so needs to be confirmed, e.g., by intracellular staining for IL-10 on T cells and DCs in the co-culture experiments. Although the decreased secretion of IL-10 in the co-cultures may hamper the anti-inflammatory effect of compounds **2** and **3**, the downregulation of IFN-γ secretion is strongly suggestive of inhibition of inflammatory Th1 response and subsequently that compounds **2** and **3** may have the potential of being a starting point for development of new anti-inflammatory drugs.

The anti-inflammatory effect of compound **4**, shown previously in a monocytic cell line [6], was confirmed in the present study as it downregulated secretion of IL-12p40 by the DCs. However, compound **4** also downregulated IL-10 production, which could interfere with the anti-inflammatory effect observed by reduced IL-12p40 secretion. This seemed to be the case as, when DCs treated with compound **4** were co-cultured with T cells, the effect of compound **4** was not anti-inflammatory as neither Th1 nor Th17 cytokines were affected. These results were unexpected and suggest that the effect of compound **4** to decrease IL-10 secretion by the DCs seems to be the determining factor, overriding the effect of downregulation of IL-12p40 and subsequently leading to a reduction in IL-10 in the co-culture of the two cell types.

Despite structural similarities of compounds **1**, **2**, **4**, and **5**, which all possess the structure of a DKP-type cyclic dipeptide, there were remarkable differences in their anti-inflammatory effects. Oxidation of the indole ring in compound **1** as compared with compound **4** caused the disappearance of the anti-inflammatory activity, indicating that the indole skeleton is important in inhibition of cytokine secretion by the DCs. Both the number and the position of double bonds on the DKP-type cyclic dipeptide may affect the anti-inflammatory activity (**2** vs. **4** vs. **5**). The disappearance of the double bond at C-8 could decrease the anti-inflammatory activity, as compound **5** did not affect cytokine secretion by the DCs whilst compounds **4** and **2** did, suggesting that the double bond at C-8 is required for the activity. However, the double bond at C-12 may be responsible for the reduction of anti-inflammatory activity when comparing compound **2** with compound **4**. Considering these observations, the bromotryptophan containing the double bond at C-8 may be important for the activity. The double bond at the *N*,*N*,*N*-trimethylethanaminium group increased the suppression of IL-12p40 production and increased the IL-10 secretion (**6** vs. **7**). Collectively, 6-bromoindole derivatives may have anti-inflammatory activity that depends on the bromotryptophan nucleus (**1**, **2**, **4**, and **5**) or the side chain at C-3 position of the 6-bromoindole (**3**, **6**, and **7**), suggesting that there may be more than one potential target site or mode of action.

The observations described above indicate that *G. barretti* is a prolific source of 6-bromoindoles with potential anti-inflammatory activities, which may be a starting point for the development of new drug s with a potential for being used in the treatment of inflammatory diseases in the future.

## 4. Materials and Methods

### 4.1. General Produres

Optical rotations were measured on a P-2000 polarimeter (Jasco, OK, USA) equipped with a 10 mm pathlength cell. UV spectra were recorded on a NanoVue^TM^ spectrophotometer (GE Healthcare Life Science, Little Chalfont, UK). ECD spectra were measured on a JASCO J-810 spectropolarimeter in quartz cells (1 or 5 mm pathlength) at 23 °C. IR spectra were measured on a Spectrum Two^TM^ FTIR spectrometer (Perkin Elmer, Waltham, MA, USA). NMR spectra were recorded on a Bruker AM-400 spectrometer (proton frequency 400.13 MHz and carbon frequency 100.62 MHz, respectively) for compounds **4**–**7** (in CD_3_OD and/or DMSO-*d*_6_) using TMS as an internal standard or a Bruker Avance 600 spectrometer (proton frequency 600.13 MHz and carbon frequency 150.76 MHz, respectively) for compounds **1** (in CD_3_OD and DMSO-*d*_6_), **2**–**3** (in CD_3_OD), and **7** (in DMSO-*d_6_*). The ^1^H NMR spectrum of compound **8** (in CDCl_3_) was recorded on a Varian Mercury 400 spectrometer and the ^13^C NMR spectrum was measured on a Varian Xsens 500 spectrometer equipped with a ^13^C{^1^H} cryoprobe at 125 MHz. High-resolution mass spectra were obtained on a Waters G1 Synapt qTOF mass spectrometer. An UHPLC system (ACQUITY UPLC Waters) was coupled in line with a qTOF mass spectrometer (Synapt G1, Waters) operating in the positive mode. HPLC was performed on a Dionex 3000 HPLC system equipped with a G1310A isopump, a G1322A degasser, a G1314A VWD detector (210 nm), a 250 × 21.2 mm Phenomenex Luna C18(2) column (5 μm), and a 250 × 4.6 mm Phenomenex Gemini-NX C18 column (5 μm). Alkaloids were detected by TLC on Merck silica gel F254 plates by immersing the plates in Dragendorff’s reagent. VLC chromatography was performed on C_18_ adsorbent (LiChroprep RP-18, 40–63 μm, Merck Inc., Darmstadt, Germany). All organic solvents were purchased from Sigma-Aldrich and were HPLC grade or the highest degree of purity.

### 4.2. Animal Materials

The sponge material *Geodia barretti* was collected in the west of Iceland (65°27.6′ N–30°46.6′ W) at 388 m depth in September 2010. The sponge was identified by Dr. Hans Tore Rapp, University of Bergen (Norway). A voucher specimen was deposited at the Department of Natural Products Chemistry, Faculty of Pharmaceutical Sciences, University of Iceland. The collected specimens (six in total) (wet weight, 1.8 kg) were immediately frozen and stored at −20 °C.

### 4.3. Extraction and Isolation

The frozen sponge material was cut into small pieces and lyophilized prior to extraction with CH_2_Cl_2_:CH_3_OH (*v*/*v*, 1:1) mixture (3 × 20 L, each for 24 h) at room temperature. The combined extracts were concentrated under *vacuum* to yield a dark gum and stored at −20 °C. The crude extract (1.8 g) was submitted to a modified Kupchan partition to yield five subextracts, namely hexane (fraction A), chloroform (fraction B), dichloromethane (fraction C), *n*-butanol (fraction D), and water (fraction E). Each fraction was analyzed by UPLC-qTOF-MS before preparative-scale isolation work commenced and the data were processed and analyzed by MassLynx programme and compared to available references in ChemSpider database [51] and SciFinder Scholar (Chemical Abstracts Service, Columbus, OH, USA). The fractions B and C showed similar patterns on TLC and the analysis of qTOF-MS data revealed similar chemical compositions. Thus, they were combined and fractionated by VLC on a C18 reversed-phase column using gradient elution of MeOH-H_2_O (10:90→ 100:0) mixtures to obtain nine fractions (F2.1−F2.9). F2.2 (750 mg) was purified by preparative HPLC (28:72:0.1 CH_3_CN-H_2_O-TFA, 8.0 mL/min) to afford compounds **3** (3.5 mg), **4** (7.3 mg), and **6** (1.5 mg) and two impure fractions (F2.2.1 and F2.2.2). F2.2.1 (78.9 mg) was purified by semi-preparative HPLC using CH_3_CN-H_2_O-TFA (27:73:0.1) to yield compounds **1** (1.8 mg) and **5** (1.2 mg). F2.2.2 (10.1 mg) was also re-chromatographed by semi-preparative HPLC (ACN-H_2_O-TFA, 31:69:1) to give compound **7** (5.3 mg). F2.4 (12.1 mg) was purified by semi-preparative HPLC (30:70:0.1 MeOH-H_2_O-TFA, 2.2 mL/min) to yield compound **2** (3.0 mg).

*Geobarrettin A* (**1**): yellow solid (1.8 mg); ${\left[\alpha \right]}_{D}^{27}$ +7 (*c* 0.2, MeOH); UV (MeOH) λ_max_ (log ε) nm: 217 (3.17), 263 (2.64); IR ν_max_ cm^−1^: 3352, 3214, 1679, 1441, 1205, 1138, 1057, 841, 802, 724; ^1^H and ^13^C NMR data, see Table 1; HRESIMS *m*/*z* 451.0728 [M + H]^+^ (calcd for C_17_H_20_^79^BrN_6_O_4_, 451.0729).

*Geobarrettin B* (**2**): yellow solid (3.0 mg); UV (MeOH) λ_max_ (log ε) nm: 231 (3.62), 270 (3.39), 289 (3.41), 372 (3.51); IR ν_max_ cm^−1^: 3297, 1677, 1438, 1206, 1139, 843, 803, 725; ^1^H and ^13^C NMR data, see Table 1; HRESIMS *m*/*z* 417.0675 [M + H]^+^ (calcd for C_17_H_18_^79^BrN_6_O_2_, 417.0675).

*Geobarrettin C* (**3**): yellow solid (3.5 mg); UV (MeOH) λ_max_ (log ε) nm: 211 (3.42), 247 (3.17), 269 (3.12), 283 (2.95); IR ν_max_ cm^−1^: 3207, 1676, 1523, 1445, 1203, 1134, 892, 802, 722; ^1^H and ^13^C NMR data, see Table 1; HRESIMS *m*/*z* 295.0440 [M]^+^ (calcd for C_13_H_16_^79^BrN_2_O^+^, 295.0446).

*Barettin* (**4**): dark yellow solid (7.3 mg); ${\left[\alpha \right]}_{D}^{25}$ −84 (*c* 0.5, MeOH), lit. ${\left[\alpha \right]}_{D}^{26}$ −32.5 (*c* 2, MeOH) [18], lit. [α] −25 (*c* 3, MeOH); HRESIMS *m*/*z* 419.0830 [M + H]^+^ (calcd for C_17_H_20_^79^BrN_6_O_2_, 419.0831); all remaining spectroscopic data are in good agreement with those previously published [9].

*8,9-Dihydrobarettin* (**5**): yellowish solid (1.2 mg); ${\left[\alpha \right]}_{D}^{25}$ −12.5 (*c* 0.096, MeOH), lit. ${\left[\alpha \right]}_{D}^{21}$ −24 (*c* 0.096, MeOH); HRESIMS *m*/*z* 421.0987 [M + H]^+^ (calcd for C_17_H_22_^79^BrN_6_O_2_, 421.0988); all remaining spectroscopic data are in good agreement with those previously published [11].

*6-Bromoconicamin* (**6**): colorless amorphous solid (1.5 mg); HRESIMS *m*/*z* 279.0495 [M]^+^ (C_13_H_16_^79^BrN_2_^+^, 279.0497); all remaining spectroscopic data are in good agreement with those previously published [14].

*l-6-Bromohypaphorine* (**7**): yellow solid (5.3 mg); [${\left[\alpha \right]}_{D}^{23}$ +36 (*c* 0.2, EtOH); HRESIMS *m*/*z* 325.0550 [M + H]^+^ (calcd for C_14_H_18_^79^BrN_2_O_2_, 325.0546); all remaining spectroscopic data are in good agreement with those previously published [46].

### 4.4. Hydrogenolysis of R-Dioxindole (***8a***) to (R)-3-Propyldioxindole (***8***)

To a solution of (*R*)-dioxindole (**8a**) (16.7 mg, 59 μmol) (92% ee, [α]_D_ −30 (*c* 0.72, MeOH); lit. (88% ee) −24.3 (*c* 0.8, MeOH) [37]) in MeOH (5.0 mL) containing Et_3_N (3 drops) was added 10% Pd-C (10 mg). The mixture was evacuated-purged with H_2_ and allowed to hydrogenate (1 atm, H_2_) for 7 h. Filtration of the mixture (Celite), followed by concentration of the eluate and passage through a short plug of silica (1:1 EtOAc-hexanes) to remove Et_3_N•HBr gave (*R*)-3-propyldioxindole (**8**) as a colorless solid. UV (CF_3_CH_2_OH) λ_max_ (log ε) nm: 208 (3.10), 257 (3.32), 268 (sh), 286 (2.74). CD (CF_3_CH_2_OH) λ_max_ (Δε) nm: 208 (+13.1), 219 (0), 232 (−10.7), 249 (0), 259 (+3.5). [α]_D_ + 96 (*c* 0.19, MeOH); + 50 (*c* 0.21, CF_3_CH_2_OH). IR (ZnSe) ν_max_ 3404, 2962, 1703, 1609, 1495, 1470, 1377, 1119, 1082, 754 cm^–1^; ^1^H NMR (CDCl_3_, 400 MHz) δ 7.37 (1H, d, *J* = 7.2 Hz, H-4), 7.33 (1H, t, *J* = 7.6 Hz, H-6), 7.10 (1H, t, *J* = 7.6 Hz, H-5), 6.83 (1H, d, *J* = 7.6 Hz, H-7), 3.19 (3H, s, *N*-Me), 1.93 (2H, m, CH_2_-1′), 1.12 (2H, m, CH_2_-2′); 0.84 (3H, t, *J* = 7.2 Hz, C-*CH_3_*-3′); ^13^C NMR (CDCl_3_, 125 MHz) δ 178.4 (C, C-2), 143.6 (C, C-7a), 130.1 (C, C-3a), 129.7 (CH, C-6), 124.0 (CH, C-5), 123.2 (CH, C-4), 108.5 (CH, C-7), 76.8 (C, C-3), 40.9 (CH_2_, C-1′), 26.3 (CH_3_, *N*-Me), 16.8 (CH_2_, C-2′), 14.2 (CH_3_, C-3′); HRESIMS *m*/*z* 228.0992 [M + Na]^+^ (calcd for C_12_H_15_NO_2_Na^+^, 228.0995) (Figures S26–S30).

### 4.5. Hydrogenolysis of Geobarrettin A (***1***) to Debromodihydrogeobarrettin A (***1a***)

A sample of compound **1** was hydrogenated (1 atm H_2_, MeOH, 20 equiv. Et_3_N) for 15 h. The mixture was filtered and concentrated to give a colorless solid (~0.4 mg) consisting of an equimolar mixture of debromodihydrogeobarrettin A (**1a**) and Et_3_N•HBr that was used, directly, for CD measurements and subsequent hydrolysis-DTA derivatization. UV-vis (CF_3_CH_2_OH) λ_max_ (log ε) nm: 207 (4.15), 269 (3.65), 281 (sh) (Figure S31); CD (CF_3_CH_2_OH) λ_max_ (Δε) nm: 208 (+13.1), 232 (−10.7), 259 (+3.5). Identical with that of (*R*)-(**8**) (Figure 3).

### 4.6. Acid Hydrolysis of Debromodihydrogeobarrettin B (***1a***)

Approximately 0.31 mg of compound **1a** was separately hydrolyzed with 6 M HCl (0.8 mL) for 15 h at 110 °C, dried under a stream of N_2_ followed by high vacuum to remove volatiles, and the resulting material subjected to further derivatization (see below).

### 4.7. Absolute Configuration of the Amino Acid of Geobarrettin A (***1***)

The determination of absolute configuration of the amino acids was performed as previously described [41]. After hydrolysis of **1a**, the residue was dissolved in acetone (50 μL) and treated with l-FDTA (25 mM in acetone, 50 μL) and 1 M NaHCO_3_ (50 μL) and heated to 80 °C for 20 min. After cooling to 23 °C, the sample was quenched with 1 M HCl (50 µL), centrifuged, and the supernatant analyzed by RP HPLC (10 μL injection, Phenomenex Luna C18, 4.6 × 250 mm, gradient elution profile (15/85–65/35 CH_3_CN-H_2_O-0.1 M NH_4_OAc-0.1% TFA, over 40 min, 0.7 mL/min). The l-FDTA derivatives were detected by UV-vis (λ_max_ 335 nm). Two peaks were eluted with retention times (ratio 19:81), coinciding with authentic l-DTA derivatives of d-Arg (25.33 min), l-Arg (26.78 min), respectively.

### 4.8. Absolute Configuration of the Amino Acid of Barettin (***4***)

A sample of barettin (**4**, 1.7 mg) was hydrogenated (1 atm H_2_, 10% Pd-C, MeOH) for 19 h. The mixture was filtered and concentrated to give debromo-8,9-dihydrobarettin (**5a**) as a colorless solid (1.4 mg) consisting of an equimolar mixture of C-3 epimers. HRESIMS *m*/*z* 343.1874 [M + H]^+^ (calcd for C_17_H_23_N_6_O_2_, 343.1877). The residue was hydrolyzed under standard conditions (6 M HCl, 15 h, see above) and the hydrolysate converted to the l-DTA derivatives and analyzed by RP HPLC as before. Two peaks, A and B, were eluted with retention times of *t*_R_ = 25.33 and 26.78 min (ratio A:B = 19:81), respectively, coinciding with authentic l-DTA derivatives of d- and l-Arg (25.33 and 26.78 min, respectively). When the same procedures were conducted on **4** without prior hydrogenation, d- and l-Arg-l-DTA derivatives were obtained in a ratio of 19:81 indicating partially racemic **4** (62% ee).

### 4.9. Maturation and Activation of DCs

DCs were developed and matured from human monocytes as previously described [52,53]. CD14^+^ monocytes were isolated from peripheral blood mononuclear cells obtained from healthy human donors using CD14 Microbeads (Miltenyi Biotech, Bergisch Gladbach, Germany). Immature DCs were obtained by culturing CD14^+^ monocytes at 5 × 10^5^ cells/mL for 7 days in the presence of IL-4 at 12.5 ng/mL and GM-CSF at 25 ng/mL (both from R&D Systems, Abingdon, England), with fresh medium and cytokines added at day 3. The immature DCs were matured and activated by culturing them at 2.5 × 10^5^ cells/mL for 24 h with IL-1β at 10 ng/mL, TNF-α at 50 ng/mL (both from R&D Systems), and lipopolysaccharide (LPS) at 500 ng/mL (Sigma-Aldrich, Munich, Germany). Pure compounds were dissolved in DMSO at the concentration of 10 μg/mL and added to the DCs at the same time as the cytokines and LPS. The final concentration of DMSO in the medium of DCs cultured with the pure compounds was 0.002% and the same concentration of DMSO was used as solvent control. In order to determine whether the effects of compounds **2**–**4** were dose-dependent, the concentrations of 2.5, 5, 7.5, 10 μg/mL were used. After 24 h the mature and activated DCs were harvested and the effects of the pure compounds on DC maturation and activation determined by measuring cytokine concentration in the culture medium by ELISA. Cell viability was determined by counting cells following staining with trypan blue and calculating the percentage of live cells.

### 4.10. Co-Culture of DCs and Allogeneic CD4^+^ T Cells

DCs matured in the presence/absence of a pure compound at 10 µg/mL or solvent control only were co-cultured with allogeneic CD4^+^ T cells at DC:T cell ratio of 1:10 (2 × 10^5^ DCs/mL:2 × 10^6^ T cells/mL) for 6 days. CD4^+^ T cells were obtained from PBMCs using CD4 Microbeads (Miltenyi Biotec). The effects of the pure compounds on the ability of the DCs to differentiate the CD4^+^ T cells were determined by measuring cytokine concentrations in the co-culture supernatants by ELISA.

### 4.11. Determination of Cytokine Concentration by ELISA

The concentrations of IL-12p40 and IL-10 in culture supernatants from DCs and of IFN-γ, IL-17 and IL-10 in supernatants from co-cultures of DCs and allogeneic CD4^+^ T cells were measured by sandwich ELISA using DuoSets from R&D Systems according to the manufacturer’s protocol. The results were expressed as secretion index (SI), which was calculated by dividing the cytokine concentration in supernatants from DCs cultured with pure compound or co-cultures of these DCs with allogeneic CD4^+^ T cells by the cytokine concentration in supernatants of DCs cultured with solvent only or co-cultures of these DCs with allogeneic CD4^+^ T cells.

### 4.12. Statistical Analysis

Data are presented as the mean values ± standard error of the mean (SEM). As the data were not normally distributed, Mann-Whitney U test or Kruskal Wallis one-way ANOVA with Tukey’s post-hoc test were used to determine statistical differences between treatments (SigmaStat 3.1, Systat Software, San Jose, CA, USA) and *p* < 0.05 was considered as statistically significant.

## 5. Conclusions

In conclusion, three new 6-bromoindole derivatives, geobarrettin A–C (**1**–**3**), and four known ones (**4**–**7**) were obtained from the marine sponge *G. barretti* collected from west of Iceland. Compounds **2**, **3** and **4** showed anti-inflammatory properties by inhibiting DC secretion of IL-12p40 with varying effects on IL-10, and the anti-inflammatory effect of compounds **2** and **3** was confirmed by inhibition of IFN-γ secretion in co-cultures of T cells and DCs matured in the presence of the compounds. It is increasingly being recognized that low-grade, subclinical inflammation is a significant pathogenic factor in many chronic diseases that have not, until now, been considered inflammatory in nature. Importantly, this includes most diseases that today are the main cause of morbidity and mortality in Western countries, such as atherosclerotic diseases, cancers, chronic pain disorders, and Alzheimer’s disease [54]. Therefore, the discovery of the two new 6-bromoindole derivatives with anti-inflammatory effects is important as they could be used in the development of treatments for diseases with inflammatory components.

## Figures and Tables

**Table 1:** NMR spectroscopic data for compounds **1**–**3** (δ in ppm, 600 MHz for ^1^H NMR and 150 MHz for ^13^C NMR).

No.	1			2		3	
	δ_H_, Mult. (*J* in Hz) ^a^	δ_H_, Mult. (*J* in Hz) ^b,c^	δ_C_, Type ^a^	δ_H_, Mult. (*J* in Hz) ^a^	δ_C_, Type ^a^	δ_H_, Mult. (*J* in Hz) ^a^	δ_C_, Type ^a^
1		10.76, br s					
2			179.3, C	7.83, s	127.6, CH	8.27, s	135.9, CH
3			78.3, C		110.0, C		116.1, C
3a			132.4, C		127.5, C		125.7, C
4	7.25, br s	7.26, d (7.9)	126.9, CH	7.61, d (8.5)	120.9, CH	8.16, d (8.5)	124.1, CH
5	7.25, br s	7.23, dd (7.9, 1.5)	127.1, CH	7.27, dd (8.5, 2.0)	124.7, CH	7.39, dd (8.5, 2.0)	127.1, CH
6			124.7, C		117.2, C		118.3, C
7	7.11, br s	7.03, d (1.5)	115.0, CH	7.62, d (2.0)	115.7, CH	7.68, d (2.0)	116.3, CH
7a			143.9, C		138.5, C		139.2, C
8	5.61, s	5.37, s	112.8, CH	7.22, s	111.0, CH		186.0, C
9			132.2, C		123.5, C	4.92, s	67.8, CH_2_
10		9.88, s					
11			167.2, C		159.3, C	3.42, s	54.9, CH_3_
12	4.28, t (6.0)	4.19, td (5.4, 1.9)	56.0, CH		130.5, C	3.42, s	54.9, CH_3_
13		8.58, d (1.9)				3.42, s	54.9, CH_3_
14			160.5, C		160.3, C		
15	1.89, m; 2.01, m	1.76, m	32.6, CH_2_	5.98, t (7.8)	114.9, CH		
16	1.67, m; 1.74, m	1.49, m; 1.55, m	24.8, CH_2_	2.57, q (7.8)	26.2, CH_2_		
17	3.24, t (6.0)	3.12, m	42.0, CH_2_	3.37, t (7.2)	41.4, CH_2_		
18		7.49, br t (5.5)					
19			158.7, C		158.8, C		
20,21		6.60–7.65, br					
3-OH		7.28, br s					

^a^ Recorded in CD_3_OD. ^b^ Recorded in DMSO-*d*_6_. ^c^ The complete assignment of proton chemical shifts was in accordance with the literature for similar DKP-indole alkaloids [10,36].

## Supplementary Materials

The following are available online at http://www.mdpi.com/1660-3397/16/11/437/s1, The 1D and 2D NMR, HRESIMS, and IR spectra of the new compounds **1**–**3**. The 1D NMR, UV, IR and HRESIMS spectra of **8**. The UV spectrum and HPLC chromatogram of L-DPT derivative of the hydrolysate **1a**.

## References

[1] Dubois R.N. (2015). The Jeremiah Metzger Lecture: Inflammation, immune modulators, and chronic disease. Trans. Am. Clin. Climatol. Assoc..

[2] Chen L., Deng H., Cui H., Fang J., Zuo Z., Deng J., Li Y., Wang X., Zhao L. (2018). Inflammatory responses and inflammation-associated diseases in organs. Oncotarget.

[3] Mayer A.M.S., Rodriguez A.D., Taglialatela-Scafati O., Fusetani N. (2017). Marine pharmacology in 2012–2013: Marine compounds with antibacterial, antidiabetic, antifungal, anti-inflammatory, antiprotozoal, antituberculosis, and antiviral activities; Affecting the immune and nervous systems, and other miscellaneous mechanisms of action. Mar. Drugs.

[4] Malve H. (2016). Exploring the ocean for new drug developments: Marine pharmacology. J. Pharm. Bioallied. Sci..

[5] Senthilkumar K., Kim S.K. (2013). Marine invertebrate natural products for anti-inflammatory and chronic diseases. Evid. Based Complement. Alternat. Med..

[6] Yuan G., Wahlqvist M.L., He G., Yang M., Li D. (2006). Natural products and anti-inflammatory activity. Asia Pac. J. Clin. Nutr..

[7] Gonzalez Y., Torres-Mendoza D., Jones G.E., Fernandez P.L. (2015). Marine diterpenoids as potential anti-inflammatory agents. Mediat. Inflamm..

[8] Keyzers R.A., Davies-Coleman M.T. (2005). Anti-inflammatory metabolites from marine sponges. Chem. Soc. Rev..

[9] Lidgren G., Bohlin L. (1986). Studies of Swedish marine organisms. 7. A novel biologically-active indole alkaloid from the sponge *Geodia baretti*. Tetrahedron Lett..

[10] Solter S., Dieckmann R., Blumenberg M., Francke W. (2002). Barettin, revisited?. Tetrahedron Lett..

[11] Sjogren M., Goransson U., Johnson A.L., Dahlstrom M., Andersson R., Bergman J., Jonsson P.R., Bohlin L. (2004). Antifouling activity of brominated cyclopeptides from the marine sponge *Geodia barretti*. J. Nat. Prod..

[12] Hedner E., Sjogren M., Hodzic S., Andersson R., Goransson U., Jonsson P.R., Bohlin L. (2008). Antifouling activity of a dibrominated cyclopeptide from the marine sponge *Geodia barretti*. J. Nat. Prod..

[13] Olsen E.K., Hansen E., Moodie W., Isaksson J., Sepcic K., Cergolj M., Svenson J., Andersen J.H. (2016). Marine AChE inhibitors isolated from *Geodia barretti*: Natural compounds and their synthetic analogs. Org. Biomol. Chem..

[14] Takahashi Y., Tanaka N., Kubota T., Ishiyama H., Shibazaki A., Gonoi T., Fromont J., Kobayashi J. (2012). Heteroaromatic alkaloids, nakijinamines, from a sponge *Suberites* sp.. Tetrahedron.

[15] Lidgren G., Bohlin L., Christophersen C. (1988). Studies of Swedish marine organisms .10. Biologically-active compounds from the marine sponge *Geodia barretti*. J. Nat. Prod..

[16] Carstens B.B., Rosengren K.J., Gunasekera S., Schempp S., Bohlin L., Dahlstrom M., Clark R.J., Goransson U. (2015). Goransson, U. Isolation, characterization, and synthesis of the barrettides: Disulfide-containing peptides from the marine sponge *Geodia barretti*. J. Nat. Prod..

[17] Hougaard L., Christophersen C., Nielsen P.H., Klitgaard A., Tendal O. (1991). The chemical-composition of species of *Geodia*, *Isops* and *Stryphnus* (Choristida, Demospongia, Porifera)–a comparative-study with some taxonomical implications. Biochem. Syst. Ecol..

[18] Johnson A.L., Bergman J., Sjogren M., Bohlin L. (2004). Synthesis of barettin. Tetrahedron.

[19] Borthwick A.D. (2012). 2,5-Diketopiperazines: Synthesis, reactions, medicinal chemistry, and bioactive natural products. Chem. Rev..

[20] Lind K.F., Hansen E., Osterud B., Eilertsen K.E., Bayer A., Engqvist M., Leszczak K., Jorgensen T.O., Andersen J.H. (2013). Antioxidant and anti-inflammatory activities of barettin. Mar. Drugs.

[21] Hedner E., Sjogren M., Frandberg P.A., Johansson T., Goransson U., Dahlstrom M., Jonsson P., Nyberg F., Bohlin L. (2006). Brominated cyclodipeptides from the marine sponge *Geodia barretti* as selective 5-HT ligands. J. Nat. Prod..

[22] Pooley J.L., Heath W.R., Shortman K. (2001). Cutting edge: Intravenous soluble antigen is presented to CD4 T cells by CD8^-^ dendritic cells, but cross-presented to CD8 T cells by CD8^+^ dendritic cells. J. Immunol..

[23] Manetti R., Gerosa F., Giudizi M.G., Biagiotti R., Parronchi P., Piccinni M.P., Sampognaro S., Maggi E., Romagnani S., Trinchieri G. (1994). Interleukin 12 induces stable priming for interferon gamma (IFN-gamma) production during differentiation of human T helper (Th) cells and transient IFN-gamma production in established Th2 cell clones. J. Exp. Med..

[24] Iyer S.S., Cheng G. (2012). Role of interleukin 10 transcriptional regulation in inflammation and autoimmune disease. Crit. Rev. Immunol..

[25] Schmitt N., Ueno H. (2015). Regulation of human helper T cell subset differentiation by cytokines. Curr. Opin. Immunol..

[26] Workman C.J., Szymczak-Workman A.L., Collison L.W., Pillai M.R., Vignali D.A. (2009). The development and function of regulatory T cells. Cell Mol. Life Sci..

[27] Kupchan S.M., Tsou G., Sigel C.W. (1973). Datiscacin, a novel cytotoxic cucurbitacin 20-acetate from *Datisca glomerata*. J. Org. Chem..

[28] Vanwagenen B.C., Larsen R., Cardellina J.H., Randazzo D., Lidert Z.C., Swithenbank C. (1993). Ulosantoin, a potent insecticide from the sponge *Ulosa ruetzleri*. J. Org. Chem..

[29] Jiang Y., Liu F.J., Wang Y.M., Li H.J. (2017). Dereplication-guided isolation of novel hepatoprotective triterpenoid saponins from *Celosiae semen* by high-performance liquid chromatography coupled with electrospray ionization tandem quadrupole-time-of-flight mass spectrometry. J. Pharm. Biomed. Anal..

[30] Wang W.G., Li A., Yan B.C., Niu S.B., Tang J.W., Li X.N., Du X., Challis G.L., Che Y., Sun H.D. (2016). LC-MS-guided isolation of penicilfuranone A: A new antifibrotic furancarboxylic acid from the plant endophytic fungus *Penicillium* sp. sh18. J. Nat. Prod..

[31] Zhang Z., Di Y.T., Wang Y.H., Zhang Z., Mu S.Z., Fang X., Zhang Y., Tan C.J., Zhang Q., Yan X.H. (2009). Gelegamines A-E: Five new oxindole alkaloids from *Gelsemium elegans*. Tetrahedron.

[32] Kamano Y., Zhang H.P., Ichihara Y., Kizu H., Komiyama K., Pettit G.R. (1995). Convolutamydine A, a novel bioactive hydroxyoxindole alkaloid from marine bryozoan *Amathia Convoluta*. Tetrahedron Lett..

[33] Wu H., Xue F., Xiao X., Qin Y. (2010). Total synthesis of (+)-perophoramidine and determination of the absolute configuration. J. Am. Chem. Soc..

[34] Ghosh D., Saravanan S., Gupta N., Abdi S.H.R., Khan N.U., Kureshy R.I., Bajaj H.C. (2014). Phosphotungstic acid as an efficient catalyst for allylation of isatins and *N*-tert-butyloxycarbonylamido sulfones under solvent-free conditions. Asian J. Org. Chem..

[35] Wei X., Henriksen N.M., Skalicky J.J., Harper M.K., Cheatham T.E., Ireland C.M., Van Wagoner R.M. (2011). Araiosamines A-D: Tris-bromoindole cyclic guanidine alkaloids from the marine sponge Clathria (Thalysias) araiosa. J. Org. Chem..

[36] Tang Y.Q., Sattler I., Thiericke R., Grabley S., Feng X.Z. (2001). Maremycins C and D, new diketopiperazines, and maremycins E and F, novel polycyclic spiro-indole metabolites isolated from *Streptomyces* sp.. Eur. J. Org. Chem..

[37] Hanhan N.V., Sahin A.H., Chang T.W., Fettinger J.C., Franz A.K. (2010). Catalytic asymmetric synthesis of substituted 3-hydroxy-2-oxindoles. Angew. Chem. Int. Ed. Engl..

[38] Salib M.N., Molinski T.F. (2018). Six trikentrin-like cyclopentanoindoles from *trikentrion flabelliforme*. Absolute structural assignment by NMR and ECD. J. Org. Chem..

[39] Sonderegger O.J., Burgi T., Limbach L.K., Baiker A. (2004). Enantio selective reduction of isatin derivatives over cinchonidine modified Pt/alumina. J. Mol. Catal. A Chem..

[40] Phyo Y.Z., Ribeiro J., Fernandes C., Kijjoa A., Pinto M.M.M. (2018). Marine natural peptides: Determination of absolute configuration using liquid chromatography methods and evaluation of bioactivities. Molecules.

[41] Salib M.N., Molinski T.F. (2017). Cyclic hexapeptide dimers, antatollamides A and B, from the ascidian *Didemnum molle*. A tryptophan-derived auxiliary for l- and d-amino acid assignments. J. Org. Chem..

[42] Kaiser K., Benner R. (2005). Hydrolysis-induced racemization of amino acids. Limnol. Oceanogr.-Meth..

[43] Albinsson B., Norden B. (1992). Excited-state properties of the indole chromophore: Electronic-transition moment directions from linear dichroism measurements: Effect of methyl and methoxy substituents. J. Phys. Chem..

[44] Shin C.G., Hayakawa M., Mikami K., Yoshimura J. (1977). Syntheses and configurational assignments of albonoursin and its three geometric isomers. Tetrahedron Lett..

[45] Shin C., Hayakawa M., Suzuki T., Ohtsuka A., Yoshimura J. (1978). α,β-Unsaturated carboxylic-acid derivatives. 13. Synthesis andconfiguration of alkyl 2-acylamino-2-alkenoates and their cyclized 2,5-piperazinedione derivatives. Bull. Chem. Soc. Jpn..

[46] Kasheverov I.E., Shelukhina I.V., Kudryavtsev D.S., Makarieva T.N., Spirova E.N., Guzii A.G., Stonik V.A., Tsetlin V.I. (2015). 6-Bromohypaphorine from marine nudibranch mollusk *Hermissenda crassicornis* is an agonist of human α7 nicotinic acetylcholine receptor. Mar. Drugs.

[47] Kondo K., Nishi J., Ishibashi M., Kobayashi J. (1994). Two new tryptophan-derived alkaloids from the Okinawan marine sponge *Aplysina* sp.. J. Nat. Prod..

[48] Corthay A. (2009). How do regulatory T cells work?. Scand. J. Immunol..

[49] Romagnani S. (1996). Th1 and Th2 in human diseases. Clin. Immunol. Immunopathol..

[50] Gee K., Guzzo C., Che Mat N.F., Ma W., Kumar A. (2009). The IL-12 family of cytokines in infection, inflammation and autoimmune disorders. Inflamm. Allergy Drug Targets.

[51] Pence H.E., Williams A. (2010). ChemSpider: An online chemical information resource. J. Chem. Educ..

[52] Freysdottir J., Sigurpalsson M.B., Omarsdottir S., Olafsdottir E.S., Vikingsson A., Hardardottir I. (2011). Ethanol extract from birch bark (*Betula pubescens*) suppresses human dendritic cell mediated Th1 responses and directs it towards a Th17 regulatory response in vitro. Immunol. Lett..

[53] Di X., Oskarsson J.T., Omarsdottir S., Freysdottir J., Hardardottir I. (2017). Lipophilic fractions from the marine sponge *Halichondria sitiens* decrease secretion of pro-inflammatory cytokines by dendritic cells and decrease their ability to induce a Th1 type response by allogeneic CD4^+^ T cells. Pharm. Biol..

[54] Iso H., Cui R., Date C., Kikuchi S., Tamakoshi A., Group J.S. (2009). C-reactive protein levels and risk of mortality from cardiovascular disease in Japanese: The JACC Study. Atherosclerosis.

