# Supplementary material for: 6-Bromoindole Derivatives from the Icelandic Marine Sponge Geodia barretti: Isolation and Anti-Inflammatory Activity
Source: Mar Drugs. 2018 Nov 8;16(11):437. doi: 10.3390/md16110437 (PMC6266195; doi:10.3390/md16110437)
Supplement: Supplementary file 1 [file marinedrugs-16-00437-s001.pdf]

Supporting Information

6-Bromoindole Derivatives from the Icelandic Marine Sponge *Geodia barretti*: Isolation and Anti-Inflammatory Activity

Xiaxia Di [1,2], Caroline Rouger [3,†], Ingibjorg Hardardottir [2,4], Jona Freysdottir [2,4], Tadeusz F. Molinski [5], Deniz Tasdemir [3,6], and Sesselja Omarsdottir [1,*]

1. Faculty of Pharmaceutical Sciences, University of Iceland, Hagi, Hofsvallagata 53, IS-107 Reykjavik, Iceland; xid1@xi.is; sesselo@hi.is
2. Department of Immunology and Centre for Rheumatology Research Landspitali-The National University Hospital of Iceland, Hringbraut, IS-101 Reykjavik, Iceland; jonaf@landspitali.is
3. GEOMAR Centre for Marine Biotechnology (GEOMAR-Biotech), Marine Natural Products Chemistry Research Unit, GEOMAR Helmholtz Centre for Ocean Research Kiel, Am Kiel-Kanal 44, Kiel 24106, Germany; caroline.rouger@u-bordeaux.fr; dtasdemir@geomar.de
4. Faculty of Medicine, Biomedical Center, University of Iceland, Vatnsmyrarvegur 16, IS-101 Reykjavik, Iceland; ih@hi.is
5. Department of Chemistry and Biochemistry and Skaggs School of Pharmacy and Pharmaceutical Sciences, University of California, San Diego, La Jolla, CA 92093, USA; tmolinski@ucsd.edu
6. Kiel University, Christian-Albrechts-Platz 4, 24118 Kiel, Germany

† Current address: Univ. de Bordeaux, UFR des Sciences Pharmaceutiques, Unité de recherche Œnologie EA 4577, USC 1366 INRA, ISVV, 210 Chemin de Leysotte, CS 50008, 33882 Villenave d'Ornon, France
* Correspondence: sesselo@hi.is; Tel.: +354-8424514

Table of Contents

Figure S1. ^{1}H NMR spectrum of geobarrettin A (**1**) (600 MHz, CD_3OD) 4

Figure S2. ^{13}C NMR spectrum of geobarrettin A (**1**) (150 MHz, CD_3OD) 5

Figure S3. HSQC spectrum of geobarrettin A (**1**) (600 MHz, CD_3OD) 6

Figure S4. HMBC spectrum of geobarrettin A (**1**) (600 MHz, CD_3OD) 7

Figure S5. ^{1}H-^{1}H COSY spectrum of geobarrettin A (**1**) (600 MHz, CD_3OD) 8

Figure S6. NOESY spectrum of geobarrettin A (**1**) (600 MHz, CD_3OD) 9

Figure S7. ^{1}H NMR spectrum of geobarrettin A (**1**) (600 MHz, DMSO-d_6) 10

Figure S8. NOESY spectrum of geobarrettin A (**1**) (600 MHz, DMSO-d_6) 11

Figure S9. HRESIMS spectrum of geobarrettin A (**1**) 12

Figure S10. IR spectrum of geobarrettin A (**1**) 13

Figure S11. ^{1}H NMR spectrum of geobarrettin B (**2**) (600 MHz, CD_3OD) 14

Figure S12. ^{13}C NMR spectrum of geobarrettin B (**2**) (150 MHz, CD_3OD) 15

Figure S13. HSQC spectrum of geobarrettin B (**2**) (600 MHz, CD_3OD) 16

Figure S14. HMBC spectrum of geobarrettin B (**2**) (600 MHz, CD_3OD) 17

Figure S15. ^{1}H-^{1}H COSY spectrum of geobarrettin B (**2**) (600 MHz, CD_3OD) 18

Figure S16. NOESY spectrum of geobarrettin B (**2**) (600 MHz, CD_3OD) 19

Figure S17. HRESIMS spectrum of geobarrettin B (**2**) 20

Figure S18. IR spectrum of geobarrettin B (**2**) 21

Figure S19. ^{1}H NMR spectrum of geobarrettin C (**3**) (600 MHz, CD_3OD) 22

Figure S20. ^{13}C NMR spectrum of geobarrettin C (**3**) (150 MHz, CD_3OD) 23

Figure S21. HSQC spectrum of geobarrettin C (**3**) in (400 MHz, CD_3OD) 24

Figure S22. HMBC spectrum of geobarrettin C (**3**) (600 MHz, CD_3OD)....25

Figure S23. ^1H-^1H COSY spectrum of geobarrettin C (**3**) (400 MHz, CD_3OD)....26

Figure S24. HRESIMS spectrum of geobarrettin C (**3**)....27

Figure S25. IR spectrum of geobarrettin C (**3**)....28

Figure S26. ^1H NMR spectrum of (*R*)-3-propyldioxindole (**8**) (400 MHz, $CDCl_3$)....29

Figure S27. ^{13}C NMR spectrum of (*R*)-3-propyldioxindole (**8**) (125 MHz, $CDCl_3$)....30

Figure S28. UV spectrum of (*R*)-3-propyldioxindole (**8**)....31

Figure S29. IR spectrum of (*R*)-3-propyldioxindole (**8**)....32

Figure S30. HRESIMS spectrum of (*R*)-3-propyldioxindole (**8**)....33

Figure S31. UV spectrum of debromodihydrogeobarrettin A (**1a**)....34

Figure S32. RP HPLC (C18) Chromatogram of L-DPT derivative of hydrolysate of debromodihydrogeobarrettin A (**1a**)....35

Figure S1. ^{1}H NMR spectrum of geobarrettin A (**1**) (600 MHz, CD_3OD)

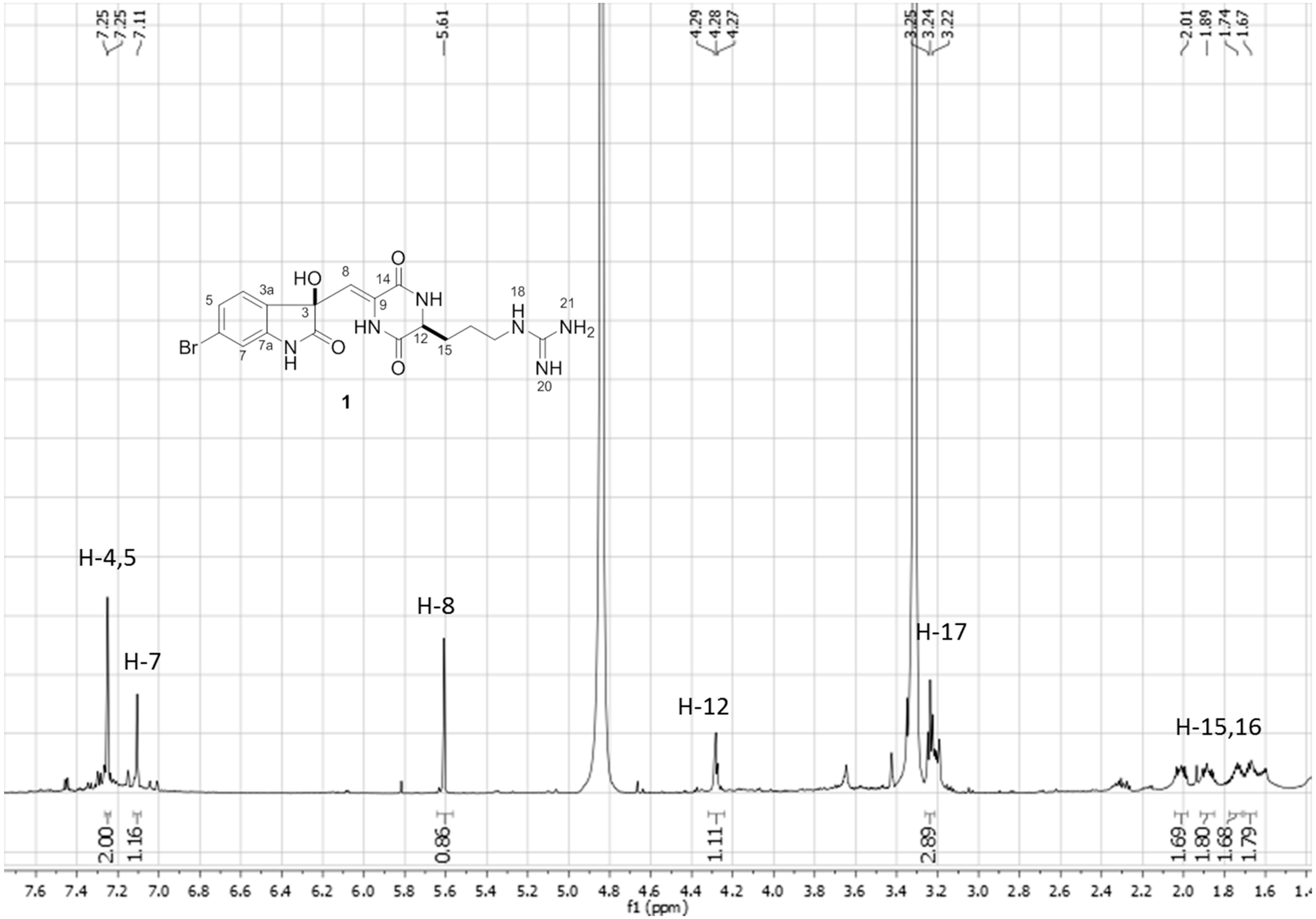

Figure S2. ^{13}C NMR spectrum of geobarrettin A (**1**) (150 MHz, CD_3OD)

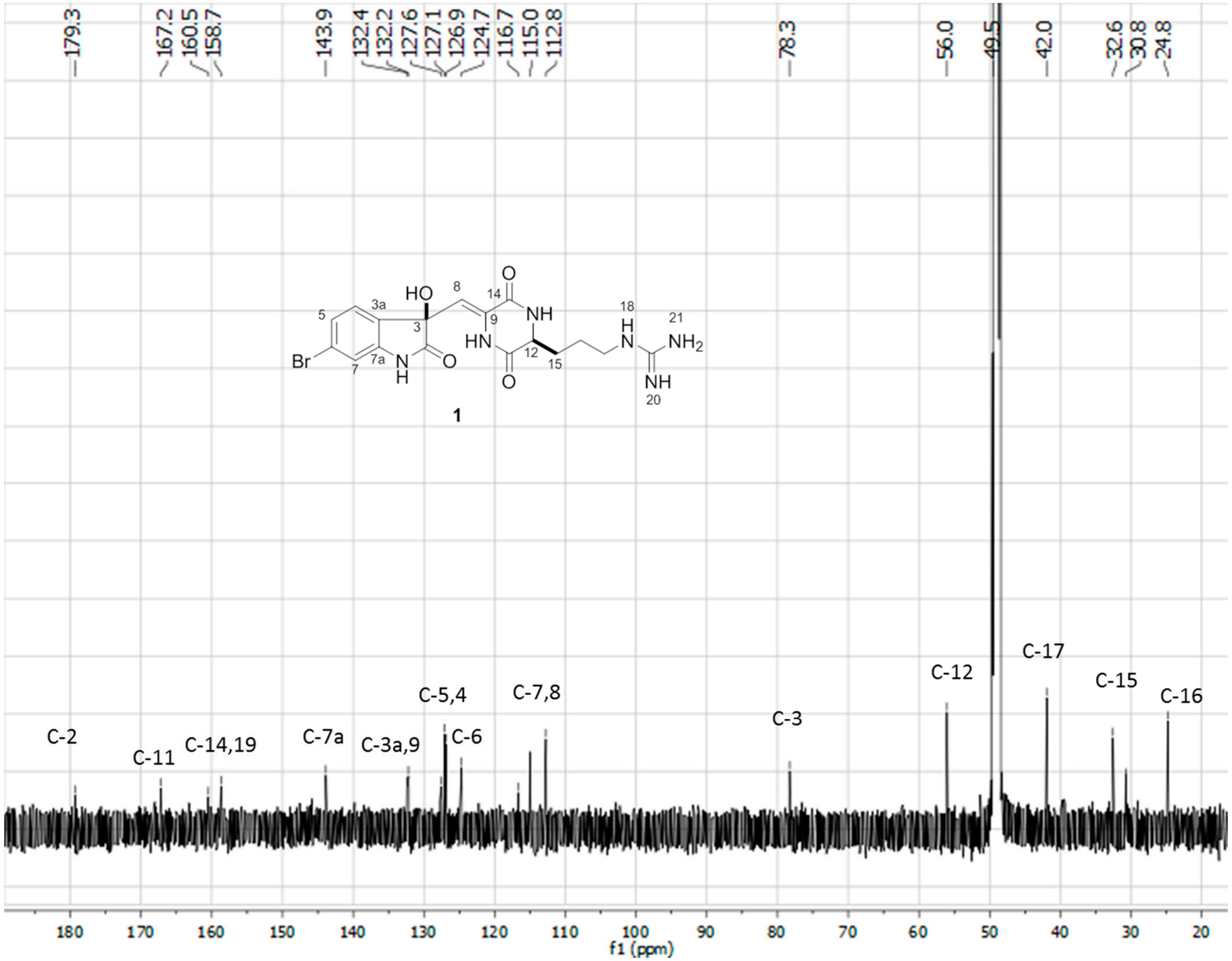

Figure S3. HSQC spectrum of geobarrettin A (**1**) (600 MHz, CD_3OD)

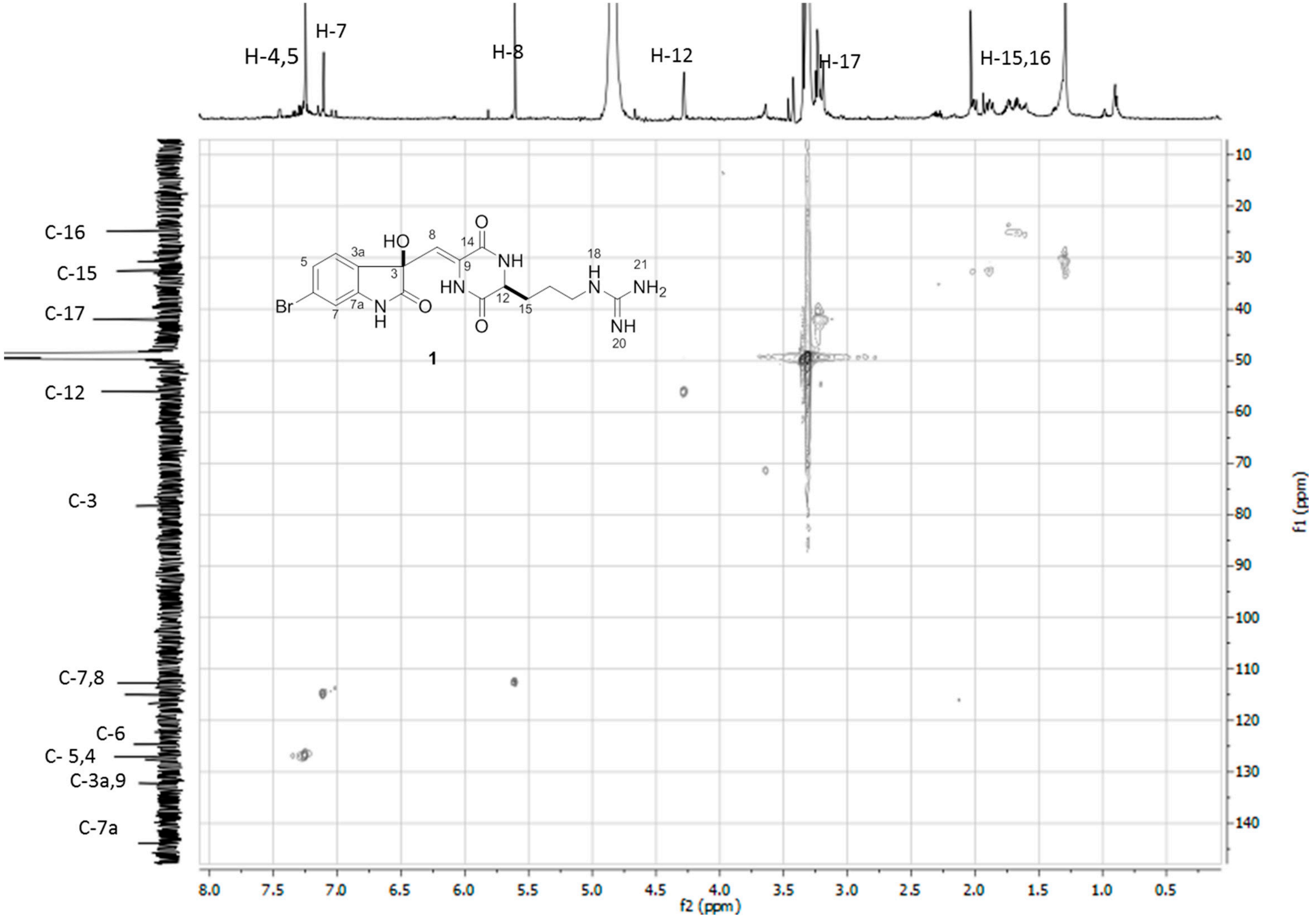

Figure S4. HMBC spectrum of geobarrettin A (**1**) (600 MHz, CD_3OD)

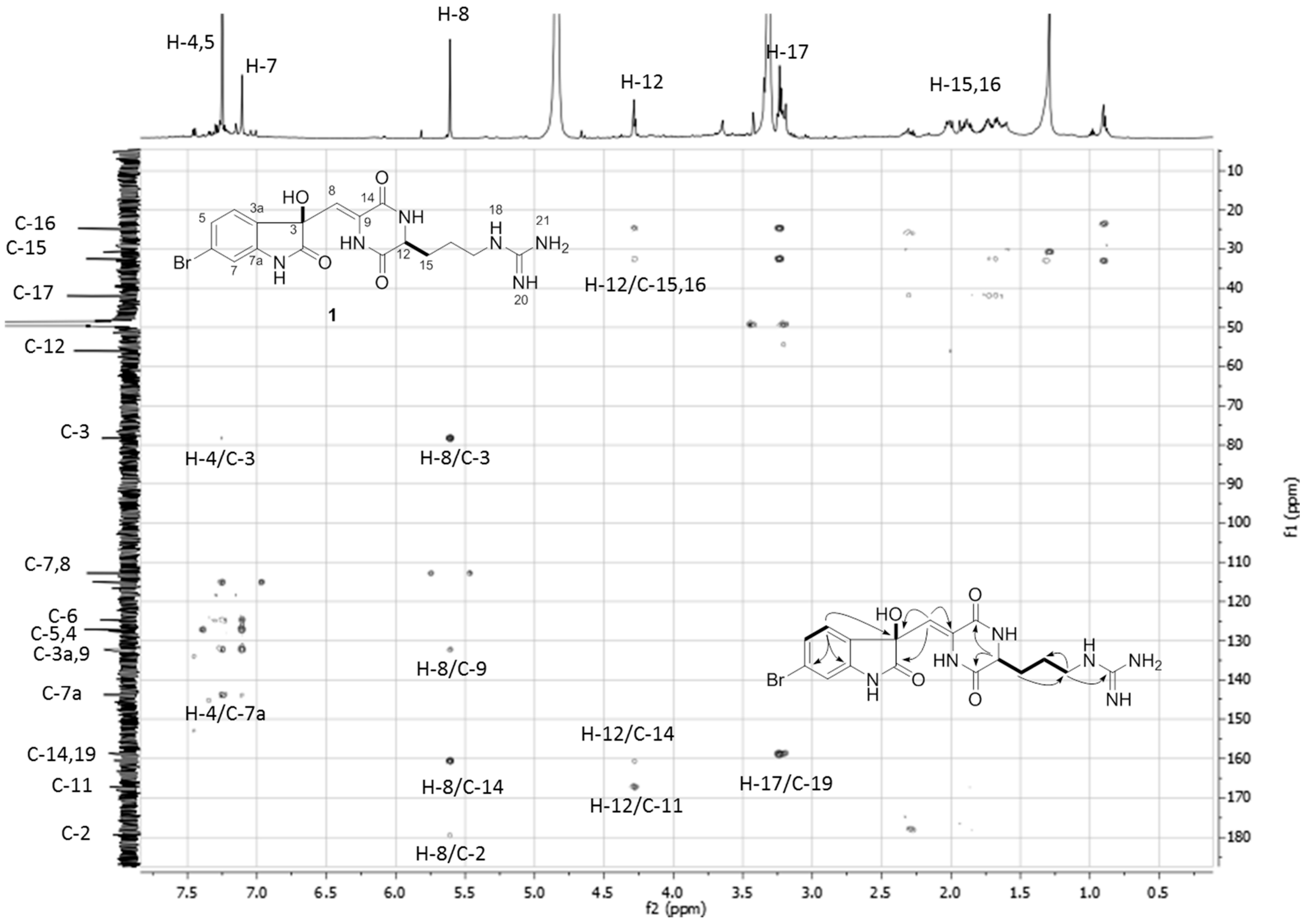

Figure S5. ^{1}H-^{1}H COSY spectrum of geobarrettin A (**1**) (600 MHz, CD_3OD)

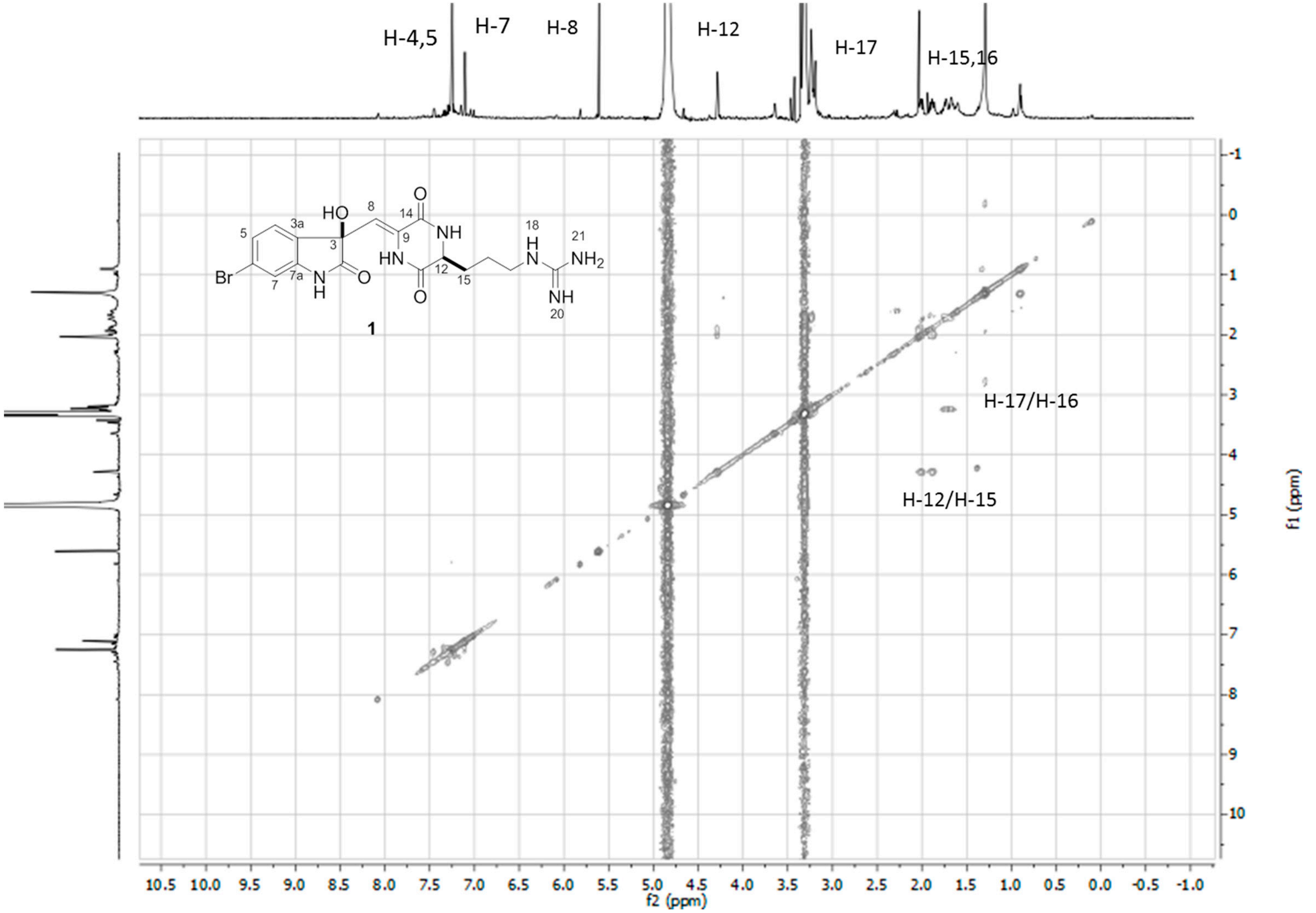

Figure S6. NOESY spectrum of geobarrettin A (**1**) (600 MHz, CD_3OD)

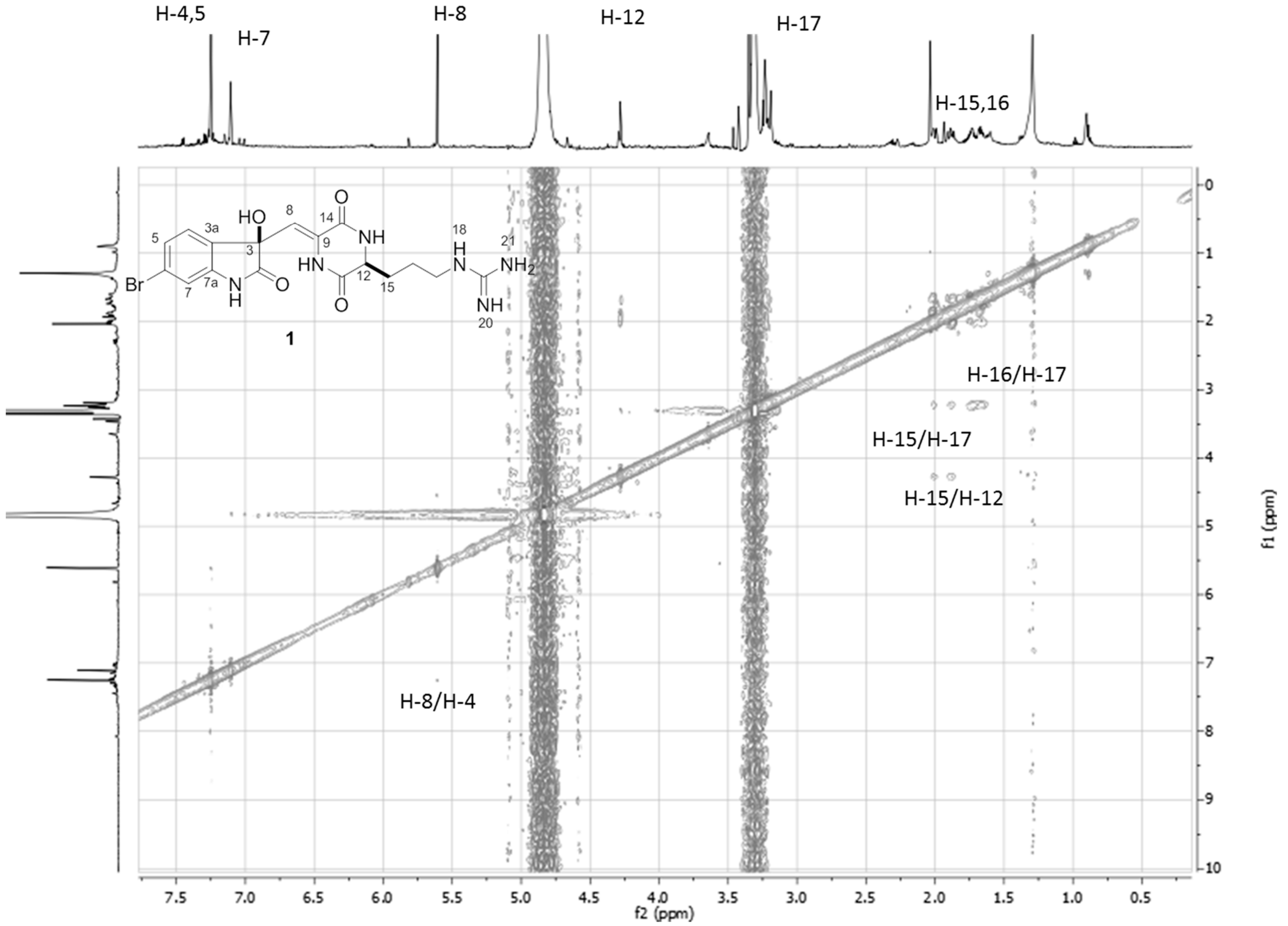

Figure S7. ^{1}H NMR spectrum of geobarrettin A (**1**) (600 MHz, DMSO-d_6)

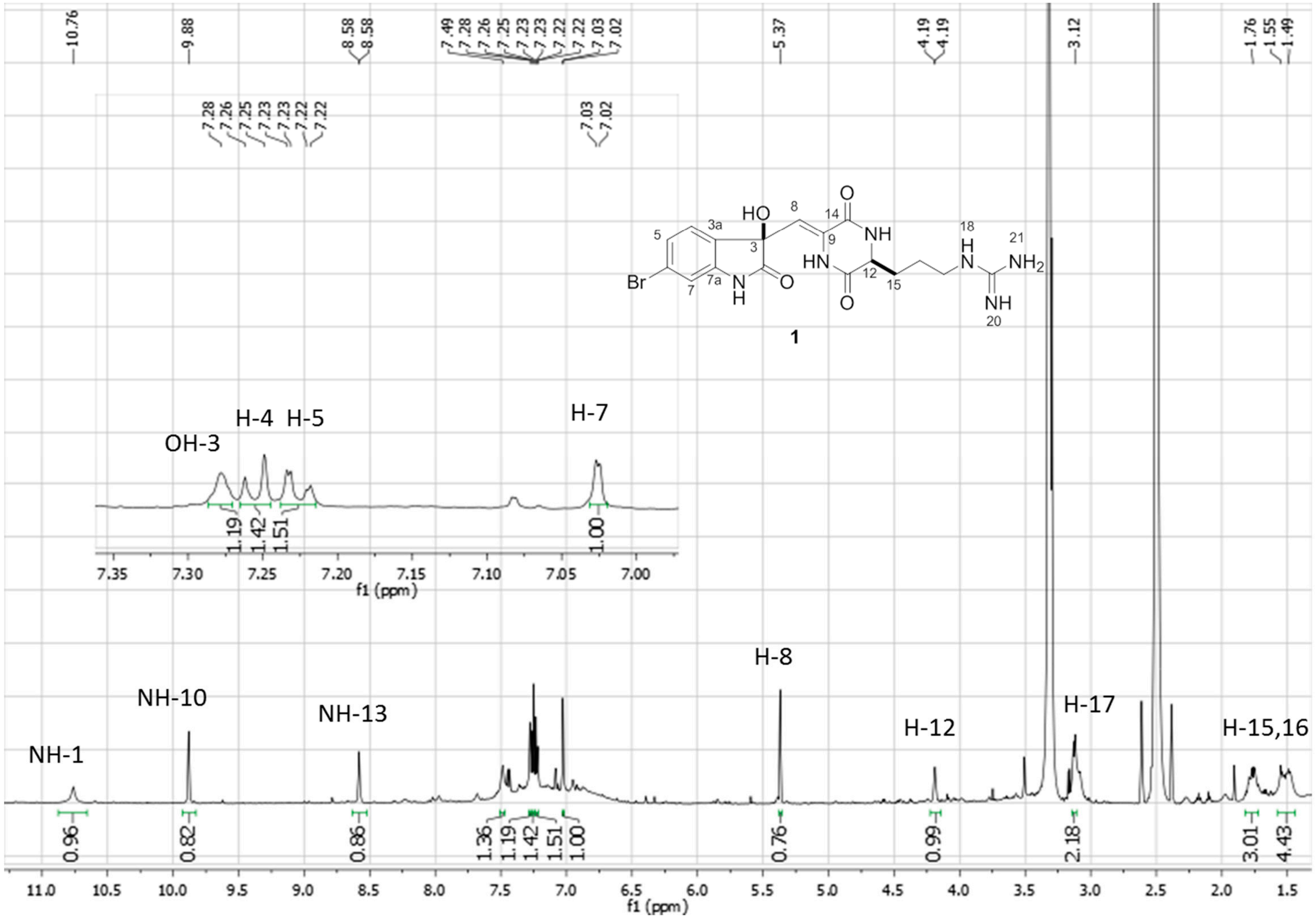

Figure S8. NOESY spectrum of geobarrettin A (**1**) (600 MHz, DMSO-d_6)

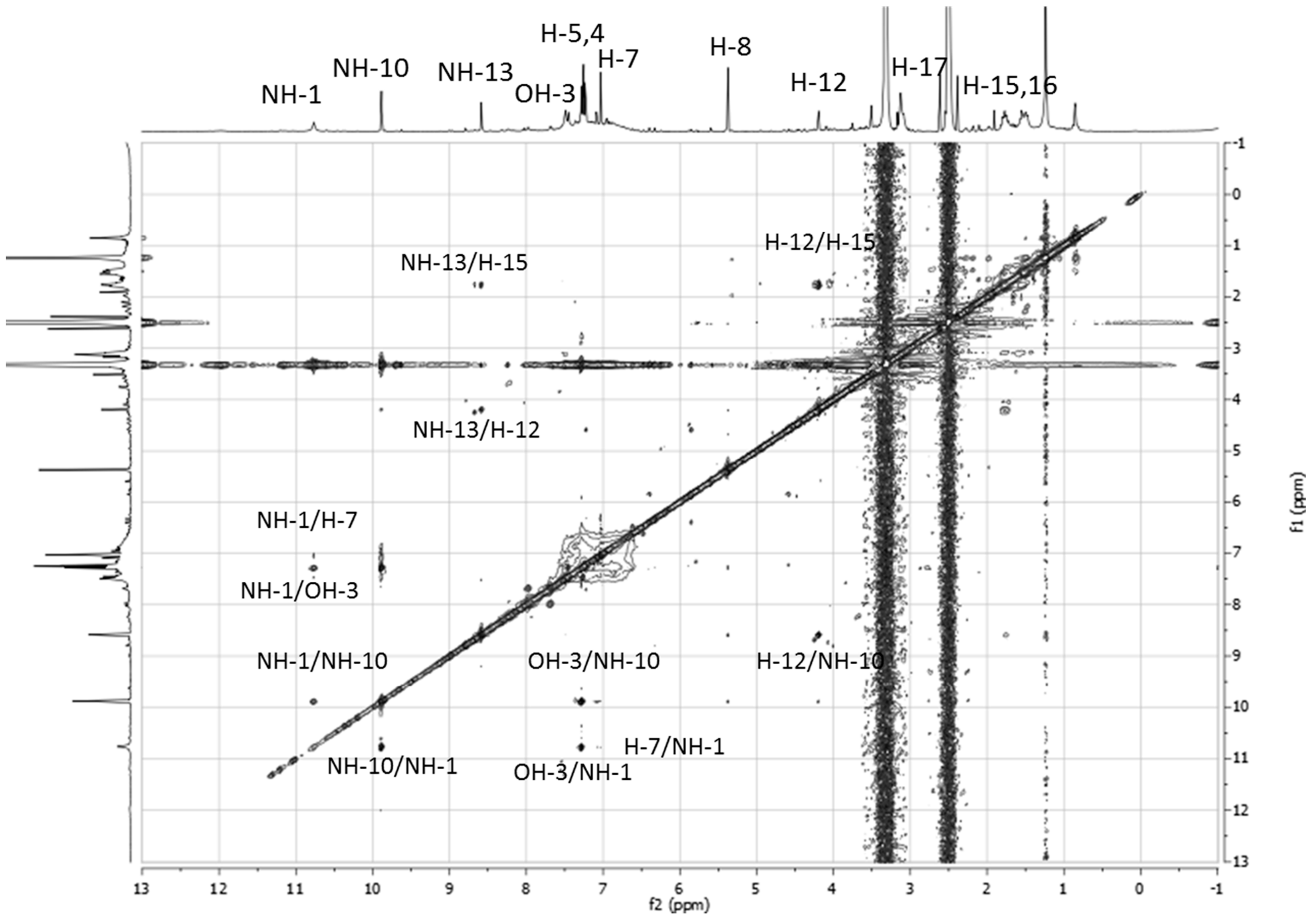

Figure S9. HRESIMS spectrum of geobarrettin A (**1**)

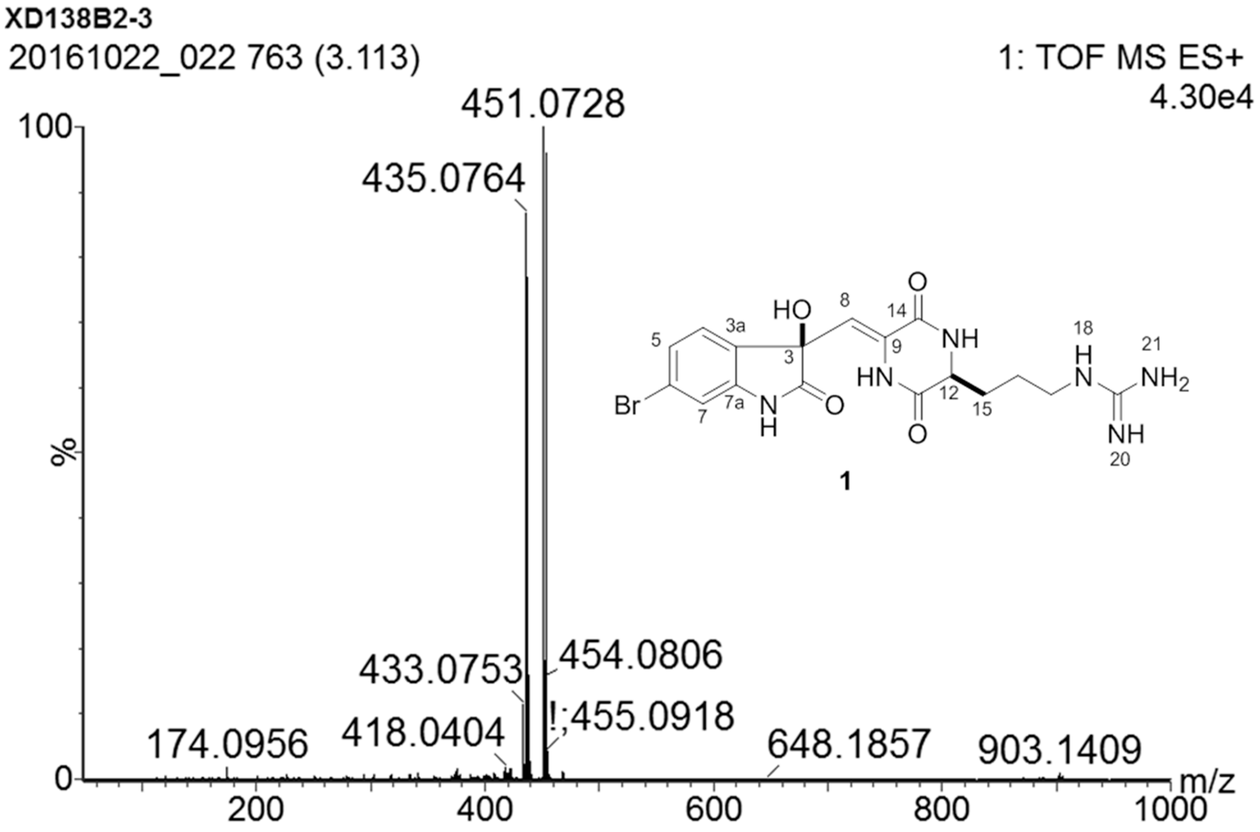

Figure S10. IR spectrum of geobarrettin A (**1**)

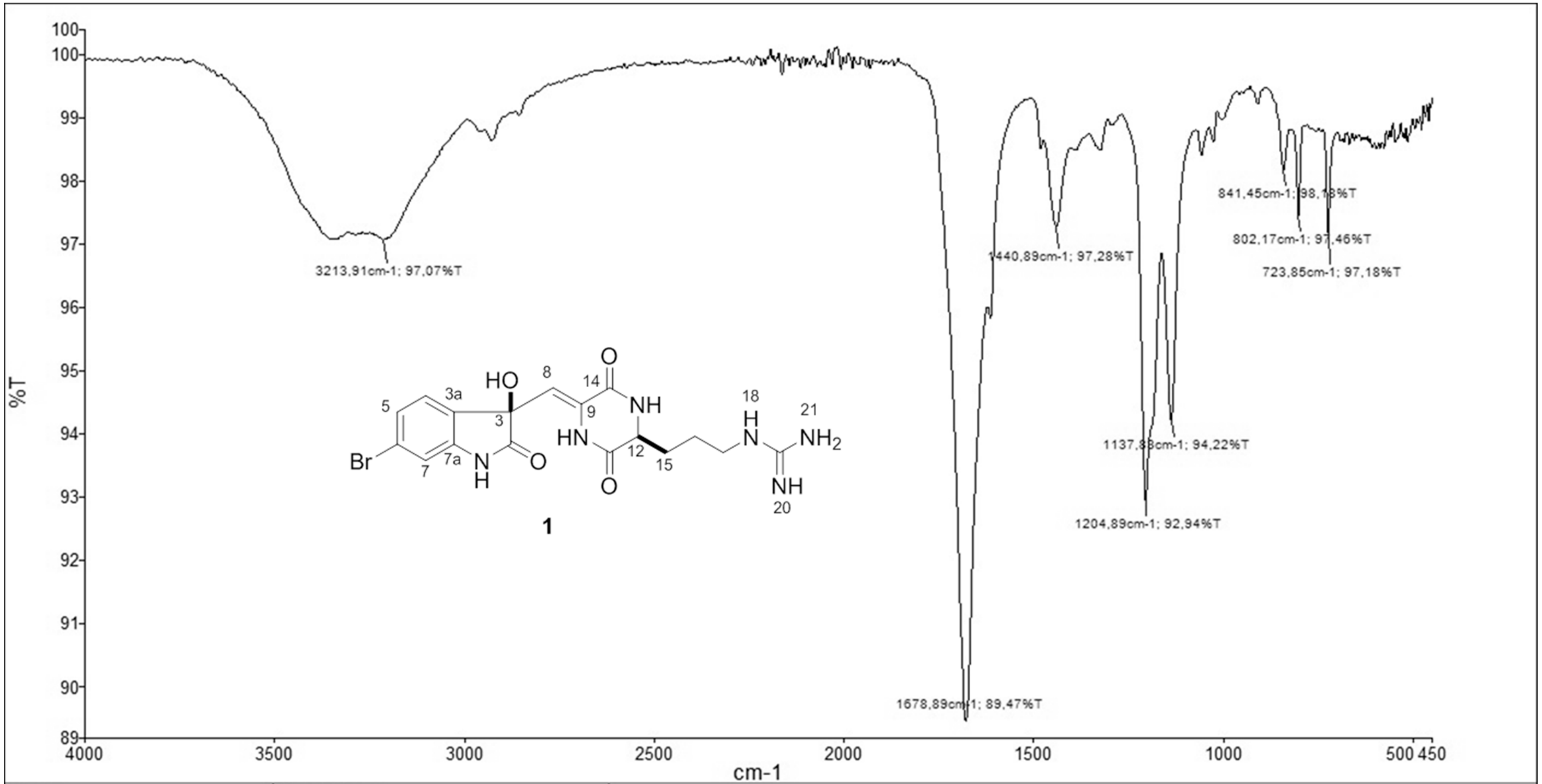

Name	Beschreibung
138_B23A1	Sample 007 By Administrator date Freitag, F...

Figure S11. ^{1}H NMR spectrum of geobarrettin B (**2**) (600 MHz, CD_3OD)

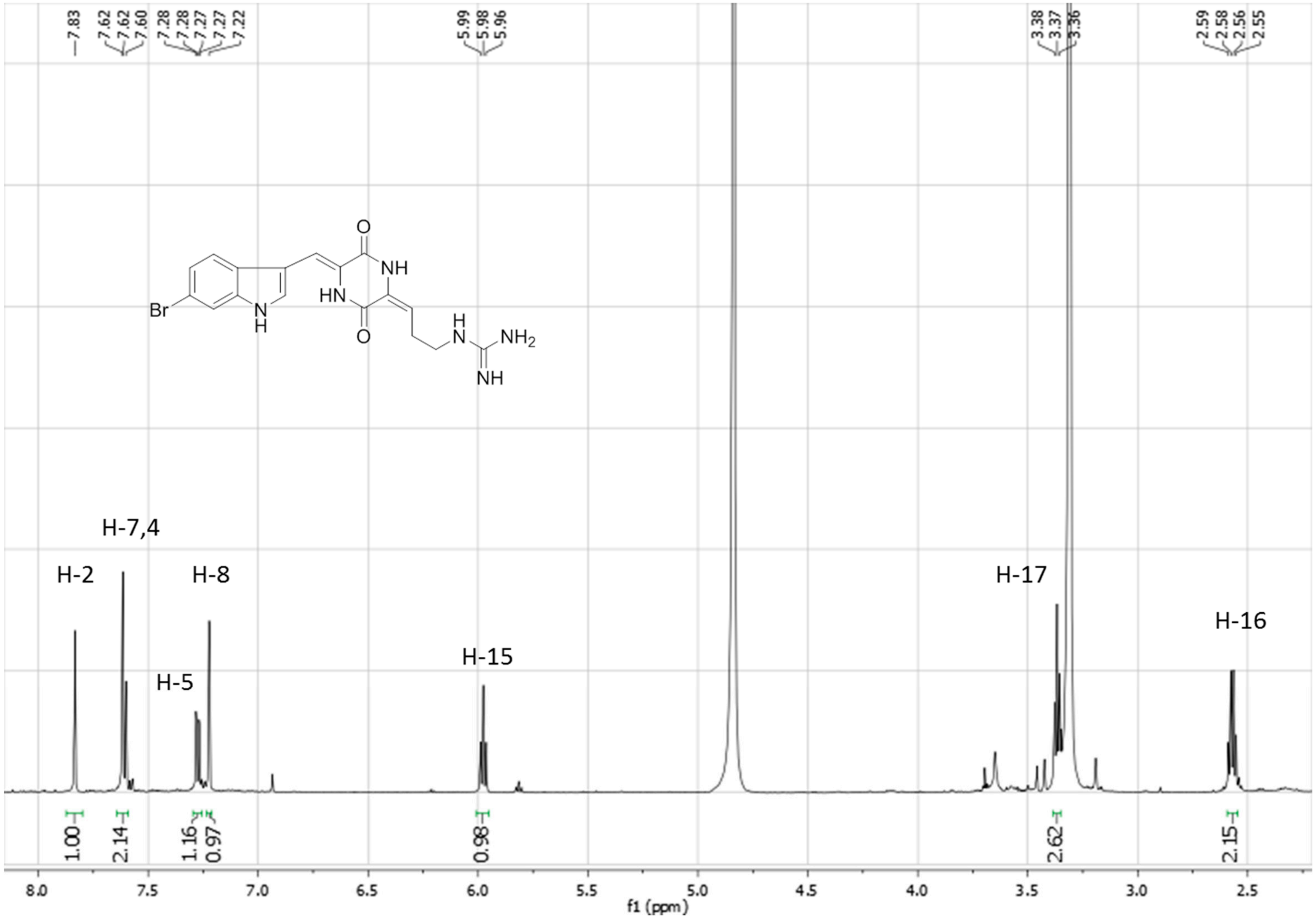

Figure S12. ^{13}C NMR spectrum of geobarrettin B (**2**) (150 MHz, CD_3OD)

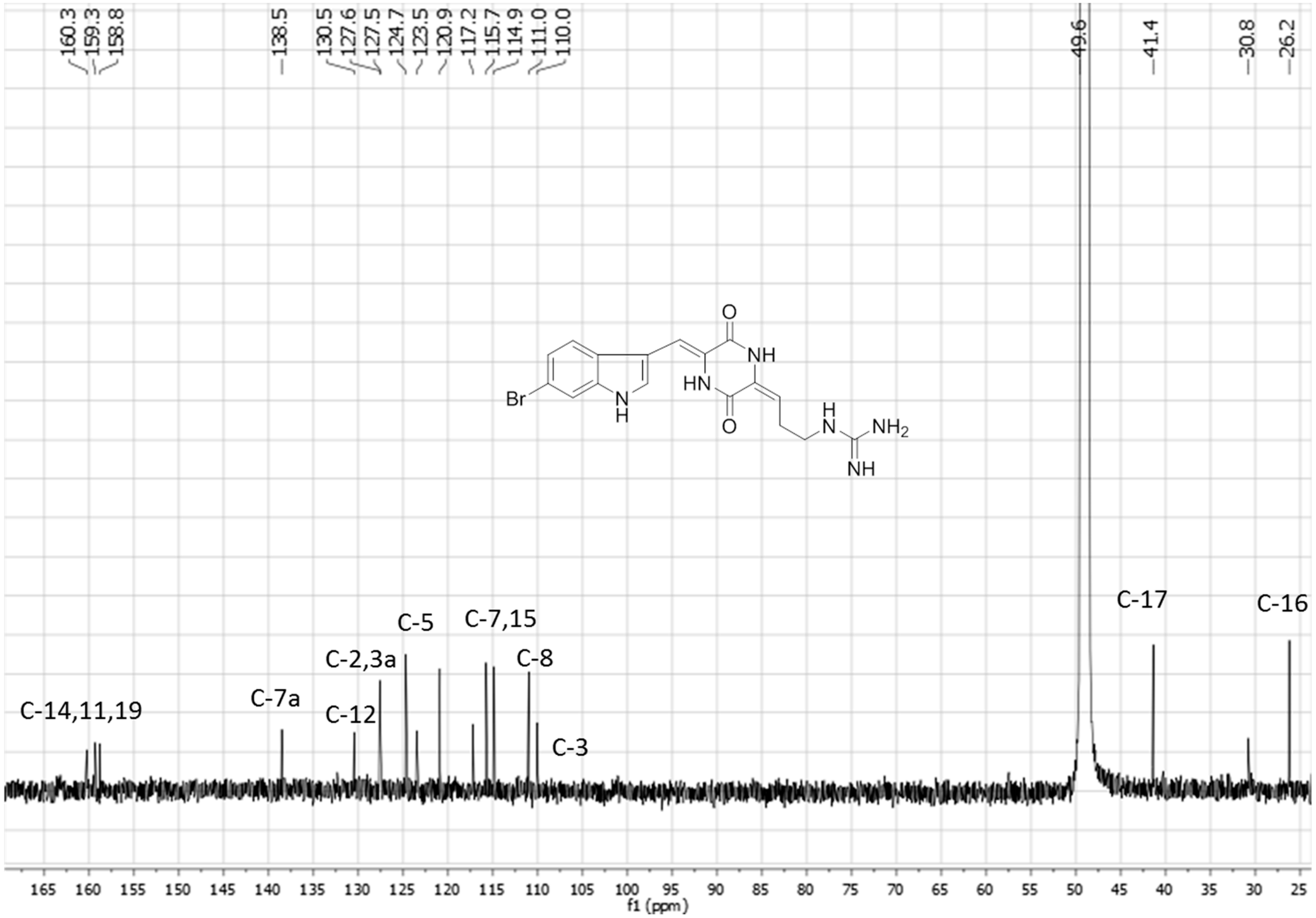

Figure S13. HSQC spectrum of geobarrettin B (**2**) (600 MHz, CD_3OD)

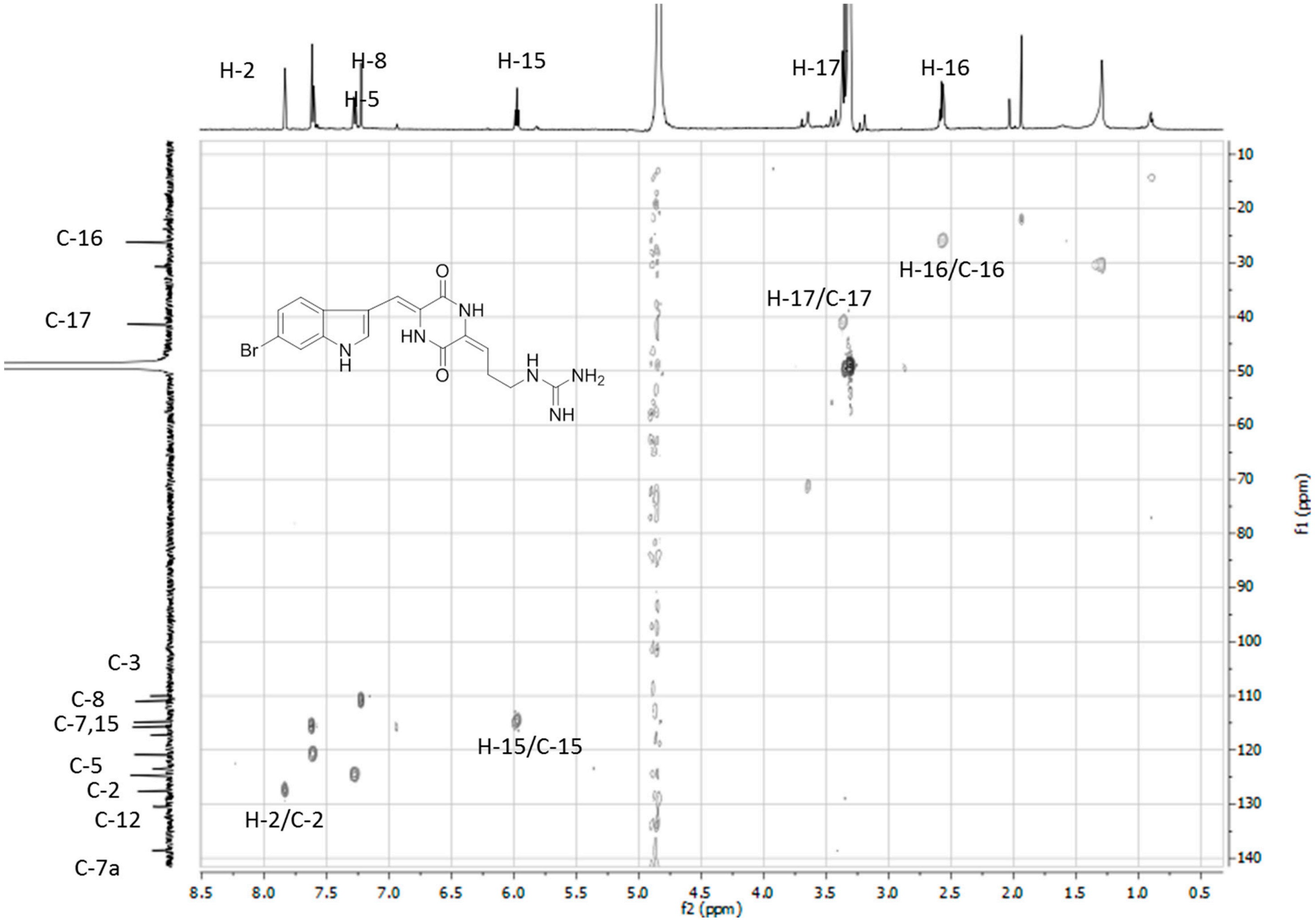

Figure S14. HMBC spectrum of geobarrettin B (**2**) (600 MHz, CD_3OD)

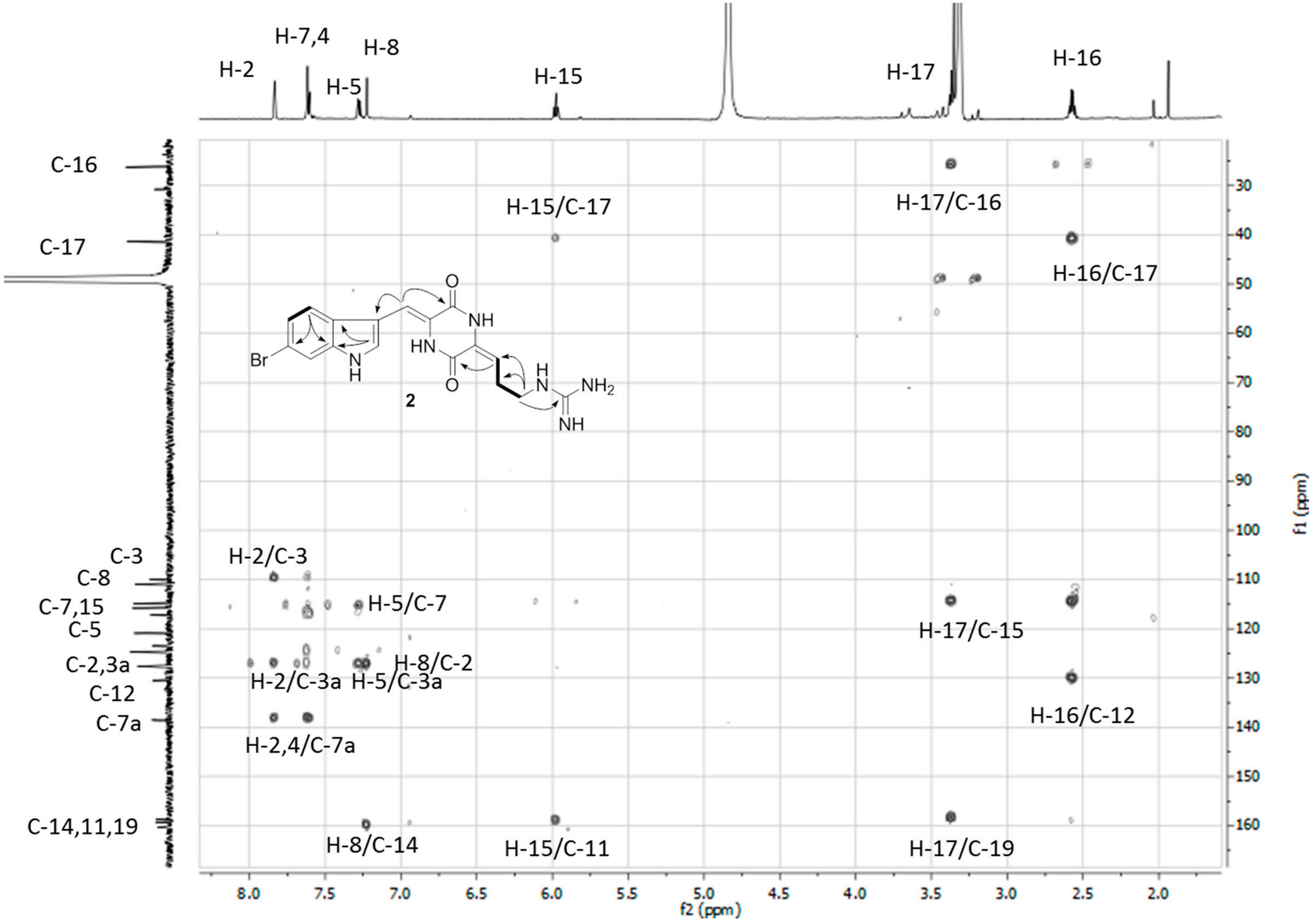

Figure S15. ^{1}H-^{1}H COSY spectrum of geobarrettin B (**2**) (600 MHz, CD_3OD)

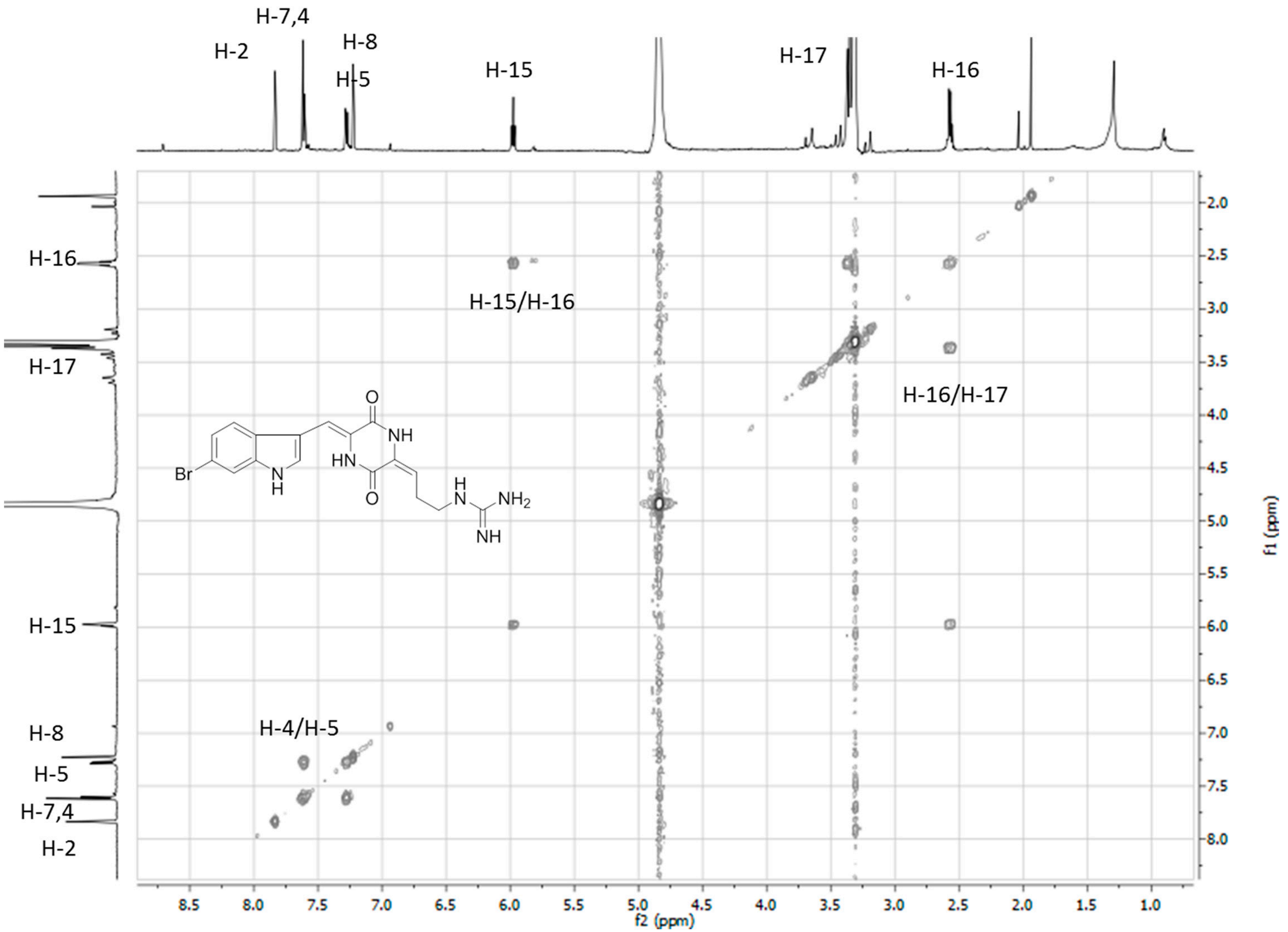

Figure S16. NOESY spectrum of geobarrettin B (**2**) (600 MHz, CD_3OD)

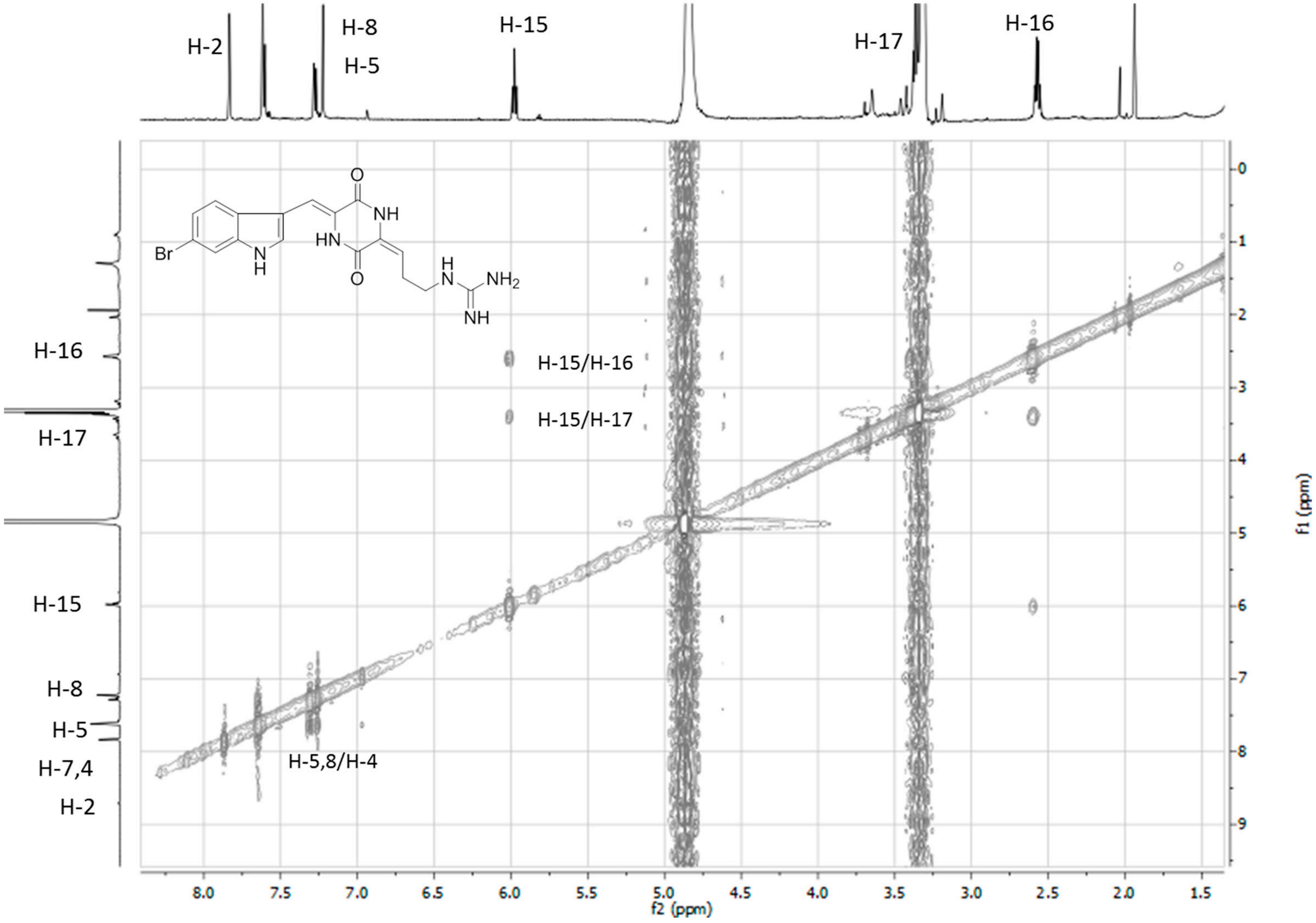

Figure S17. HRESIMS spectrum of geobarrettin B (**2**)

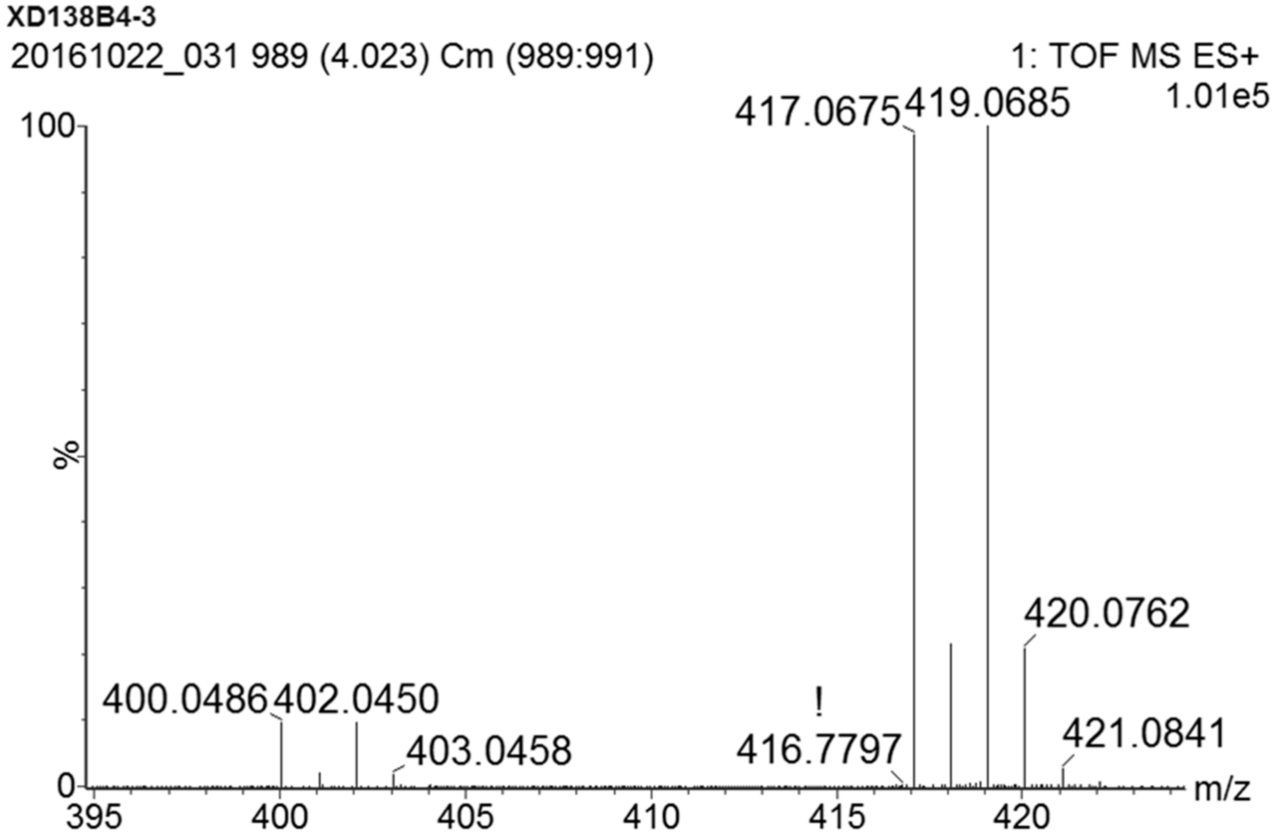

Figure S18. IR spectrum of geobarrettin B (**2**)

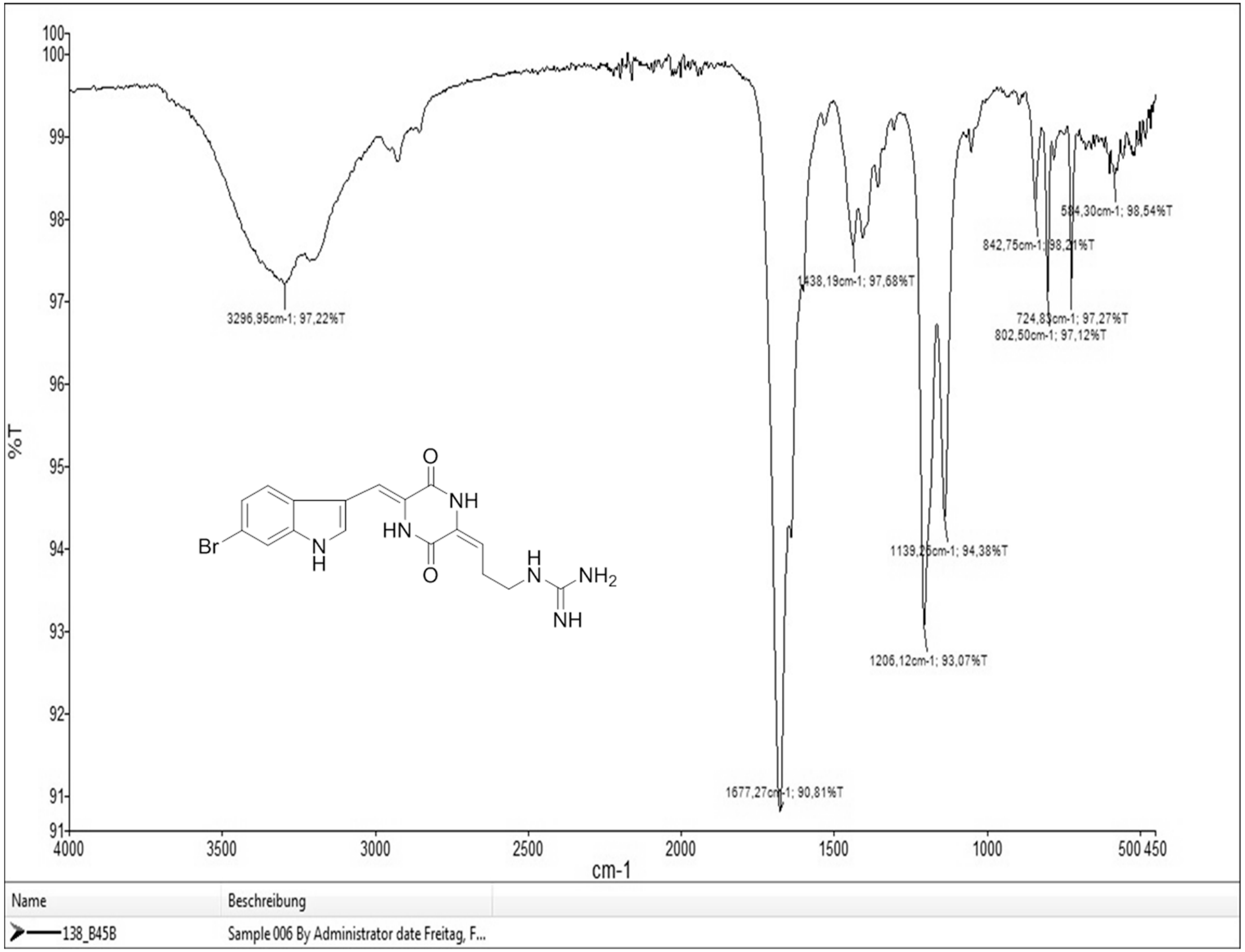

Figure S19. ¹H NMR spectrum of geobarrettin C (**3**) (600 MHz, CD_3OD)

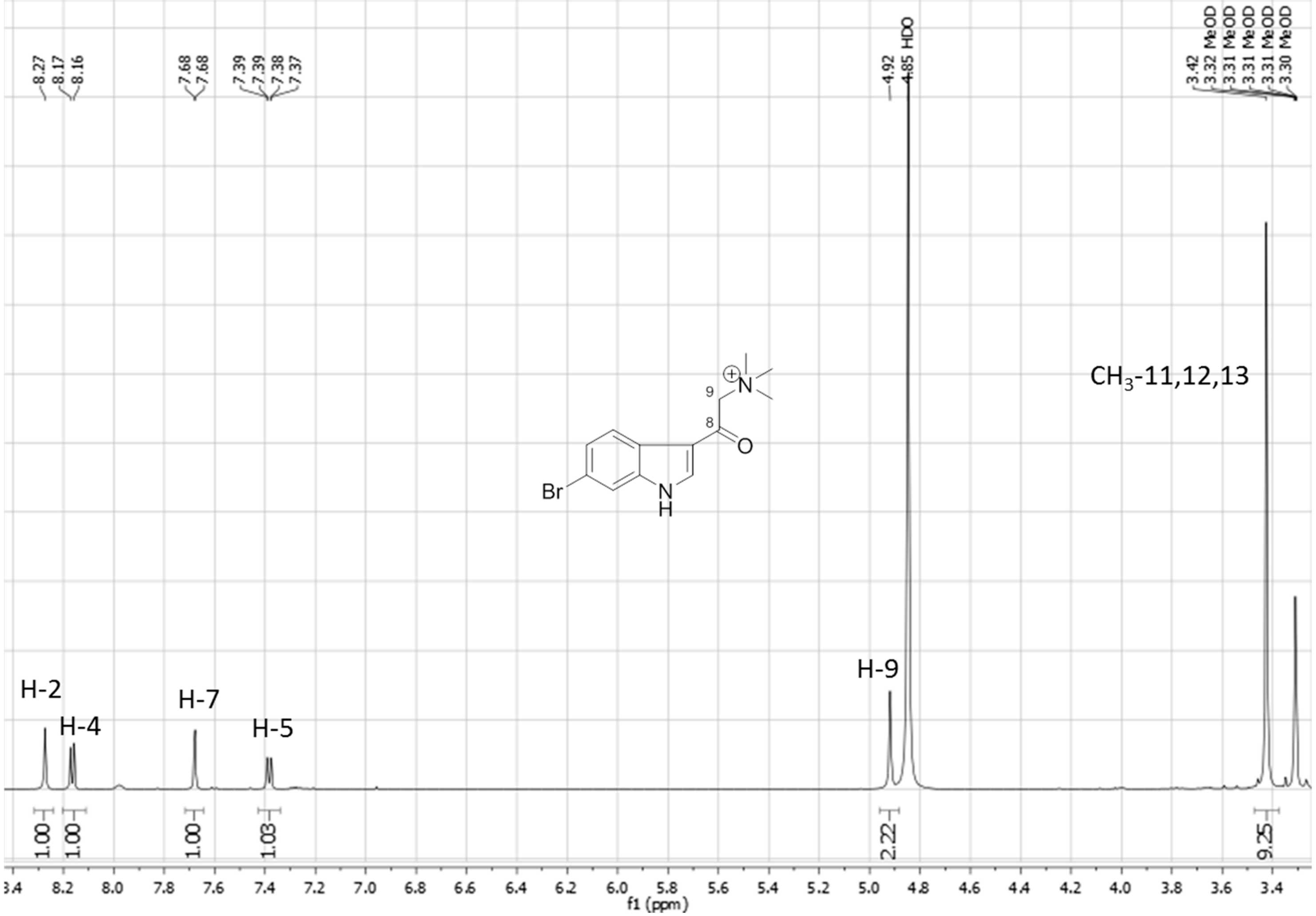

Figure S20. ^{13}C NMR spectrum of geobarrettin C (**3**) (150 MHz, CD_3OD)

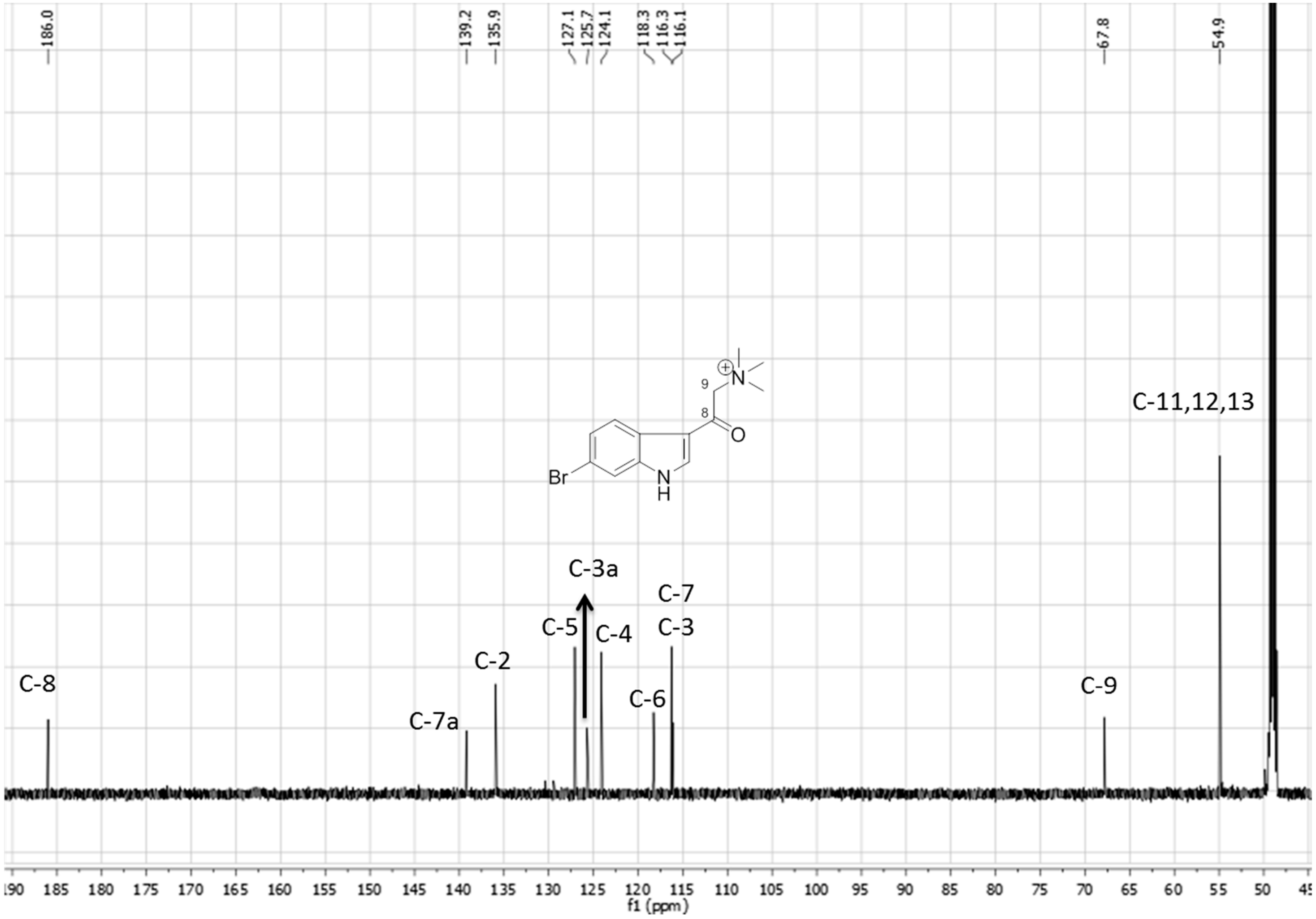

Figure S21. HSQC spectrum of geobarrettin C (**3**) in (400 MHz, CD_3OD)

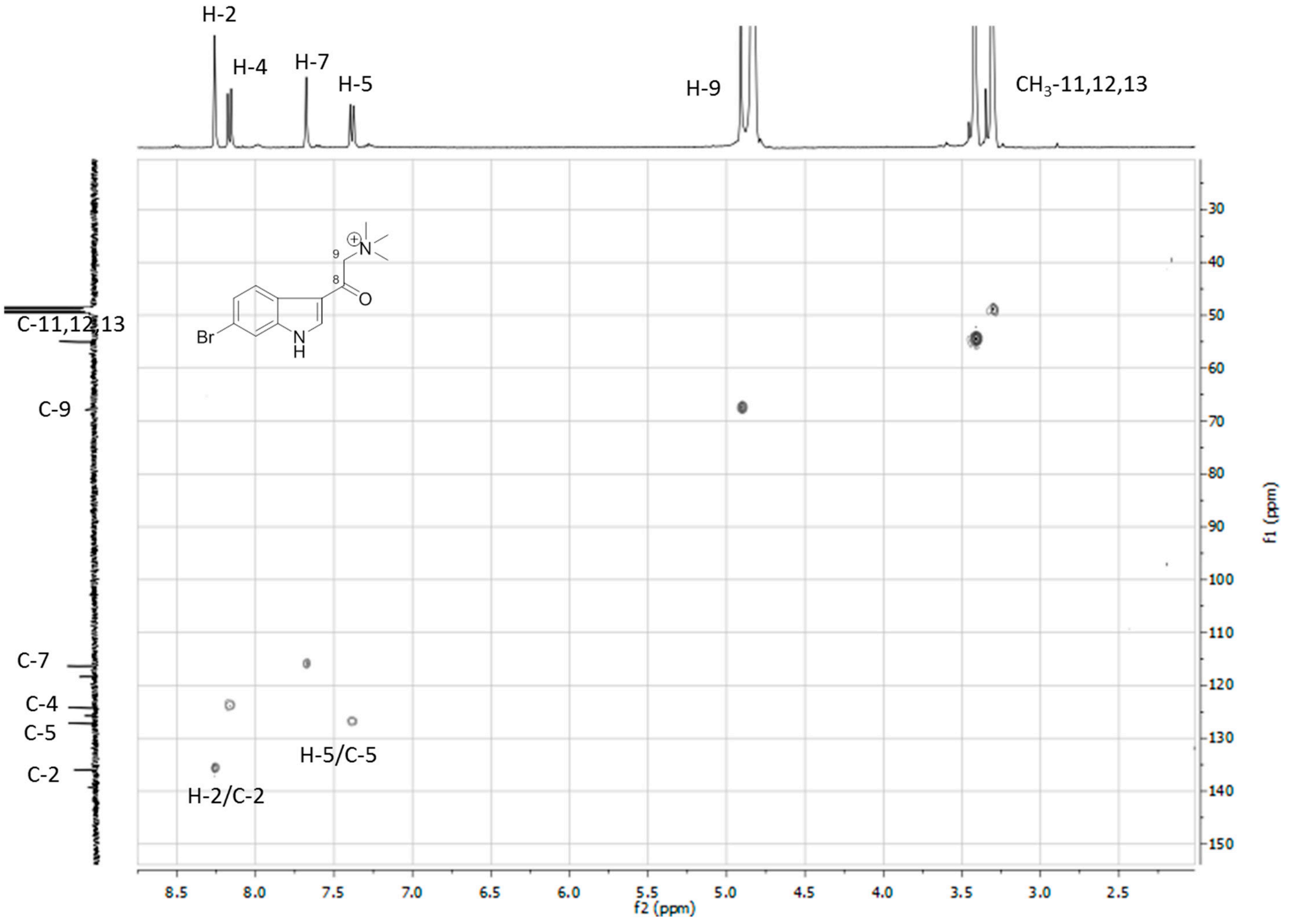

Figure S22. HMBC spectrum of geobarrettin C (**3**) (600 MHz, CD₃OD)

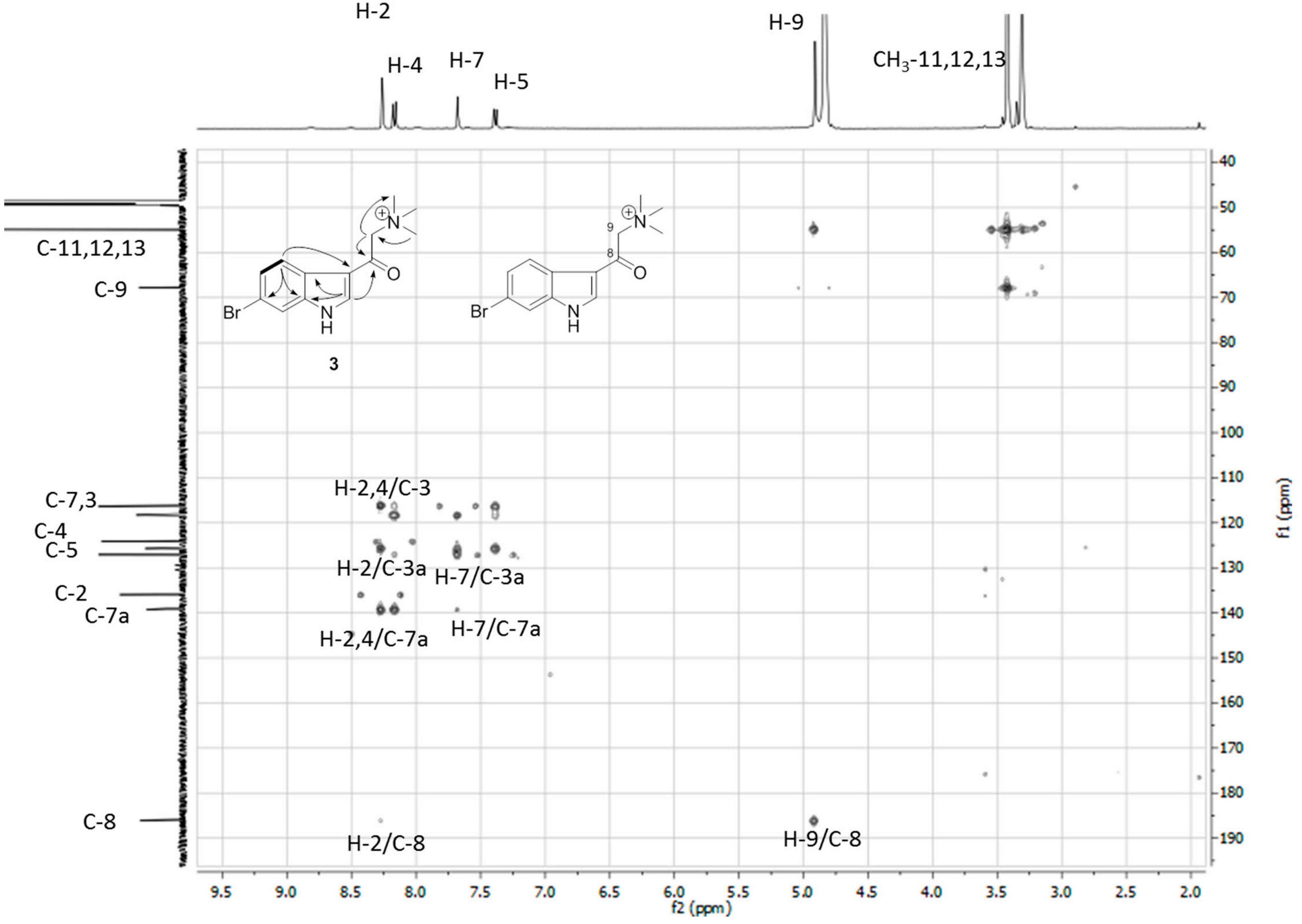

Figure S23. ¹H-¹H COSY spectrum of geobarrettin C (**3**) (400 MHz, CD_3OD)

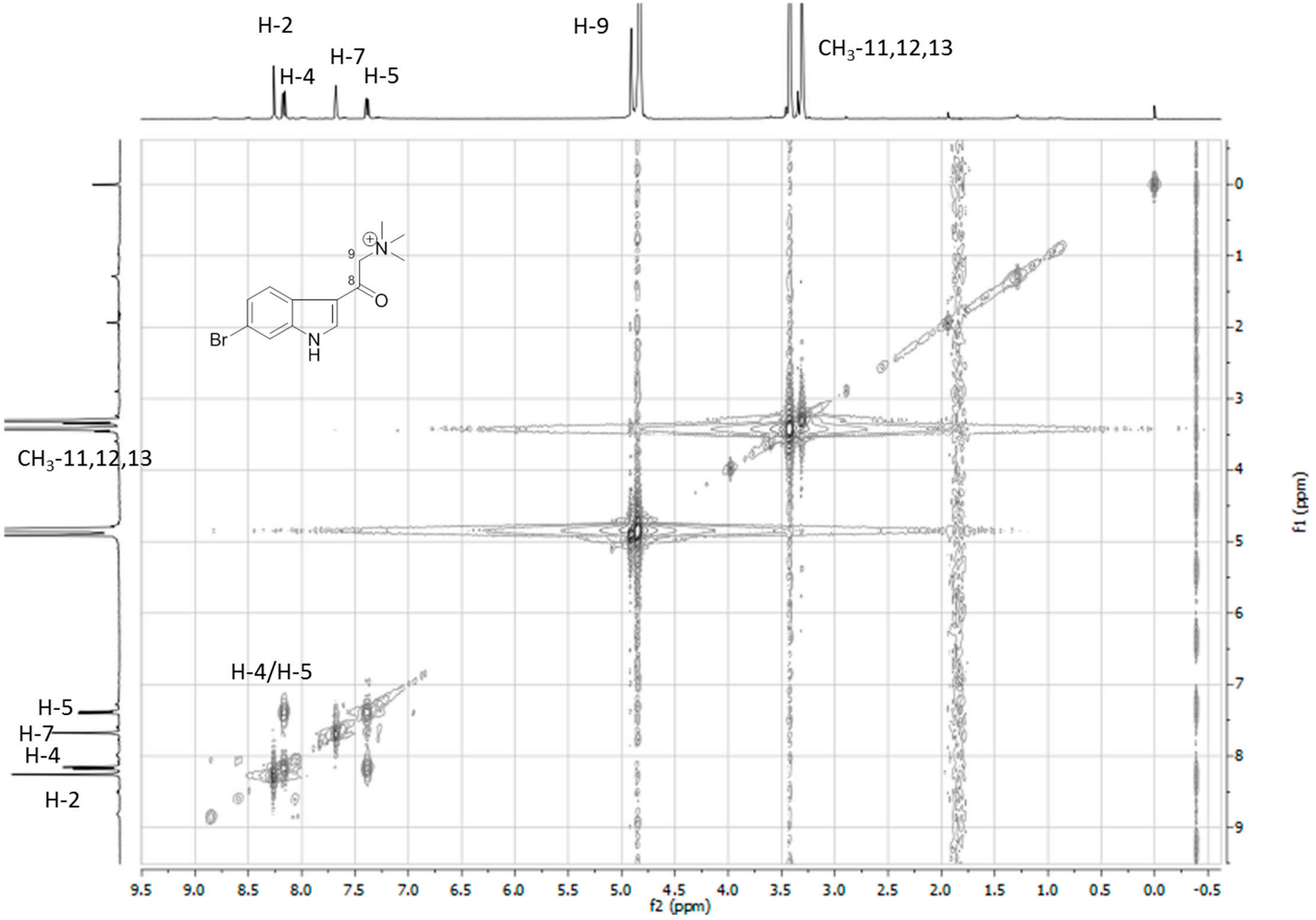

Figure S24. HRESIMS spectrum of geobarrettin C (**3**)

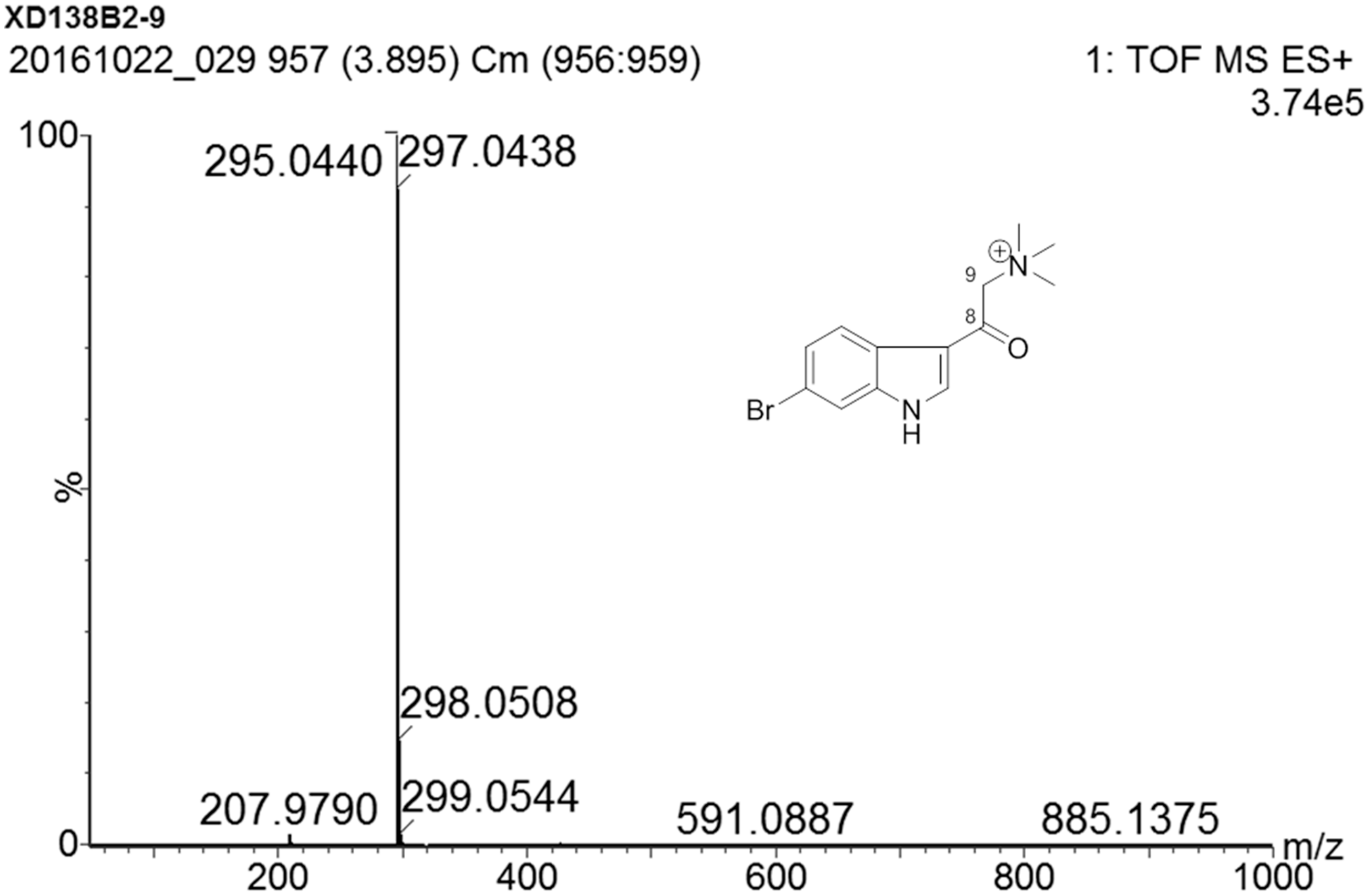

Figure S25. IR spectrum of geobarrettin C (**3**)

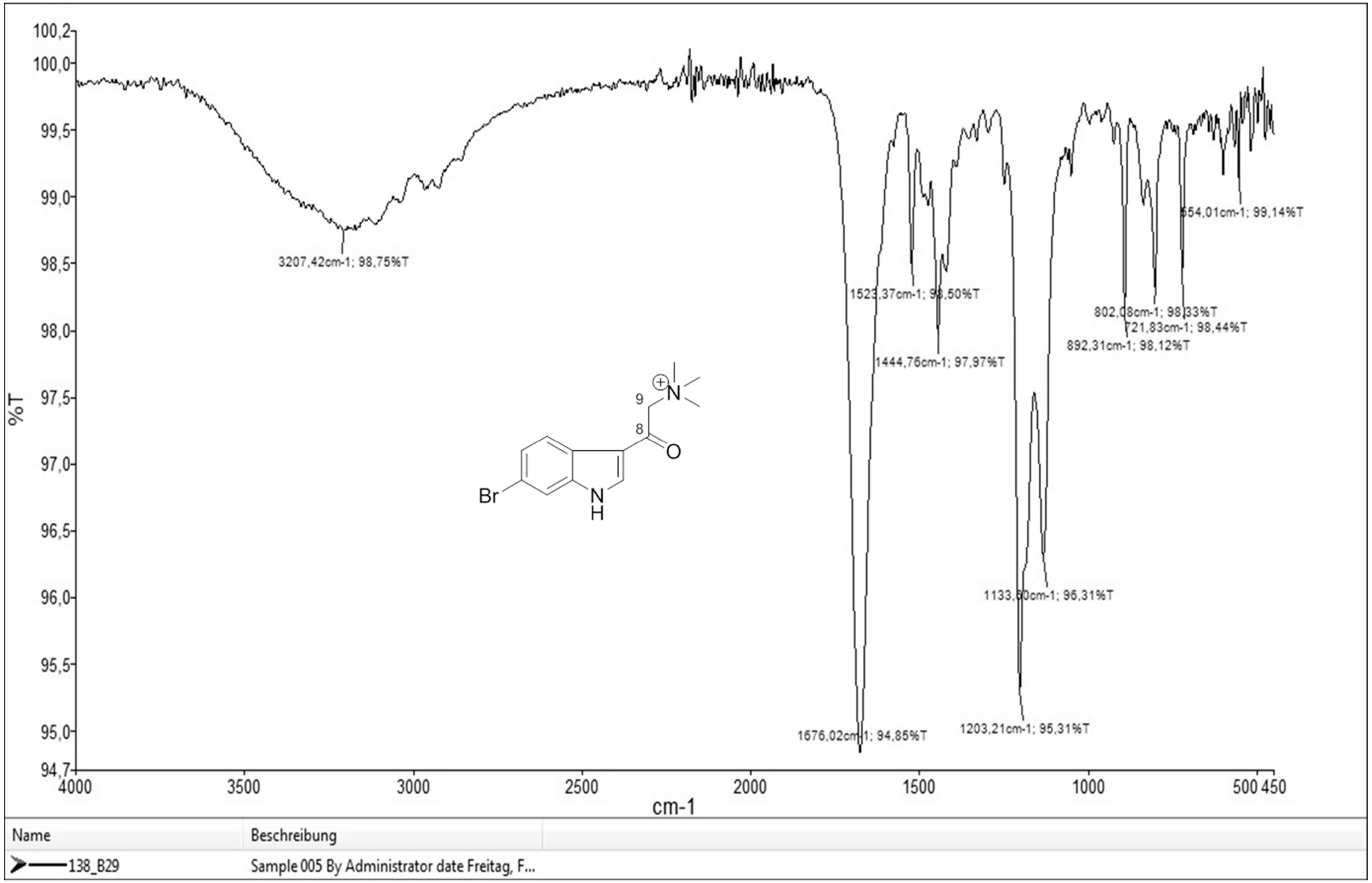

Figure S26. ^{1}H NMR spectrum of (*R*)-3-propyldioxindole (**8**) (400 MHz, $CDCl_3$)

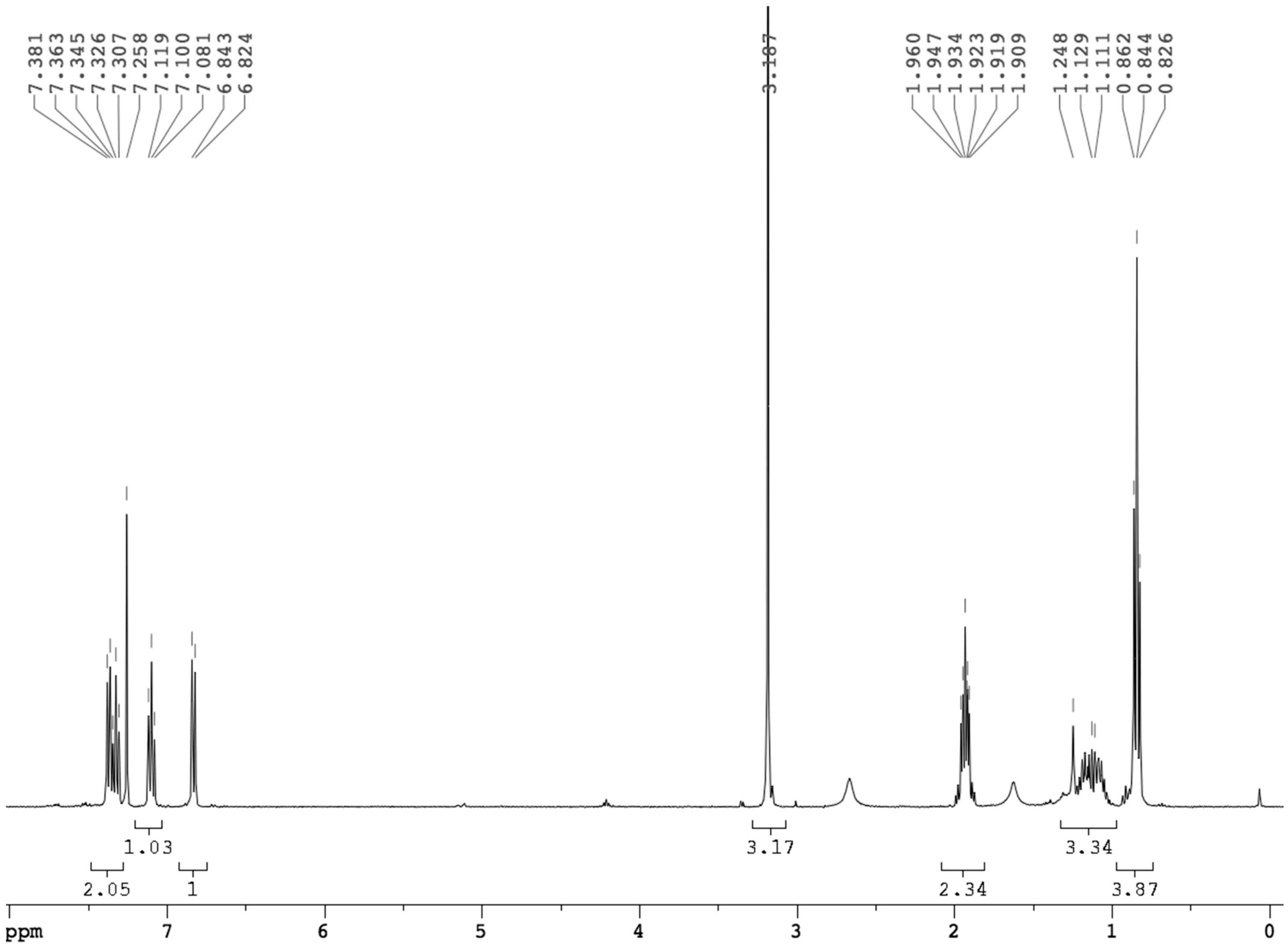

Figure S27. ^{13}C NMR spectrum of (*R*)-3-propyldioxindole (**8**) (125 MHz, $CDCl_3$)

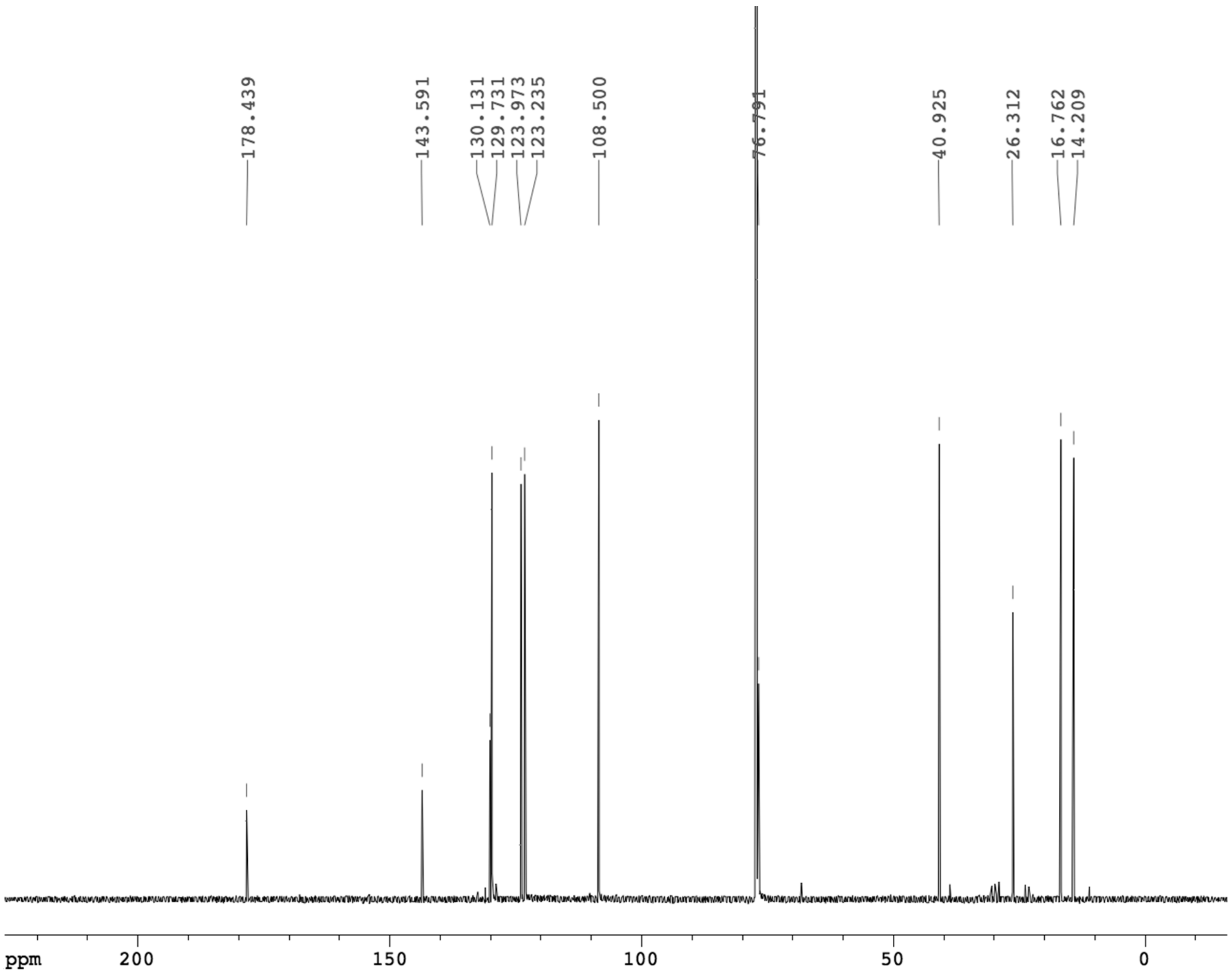

Figure S28. UV spectrum of (*R*)-3-propyldioxindole (**8**)

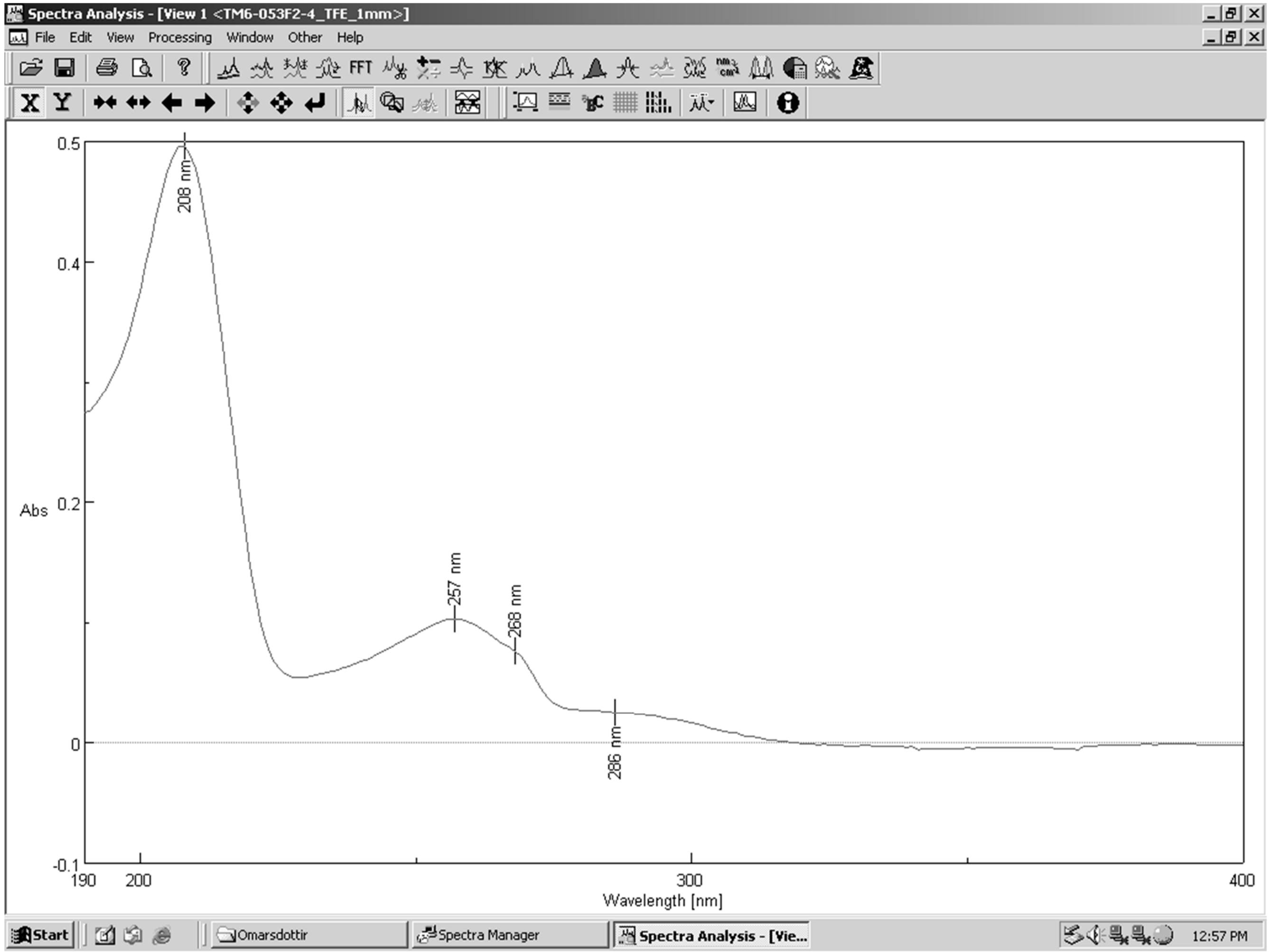

Figure S29. IR spectrum of (*R*)-3-propyldioxindole (**8**)

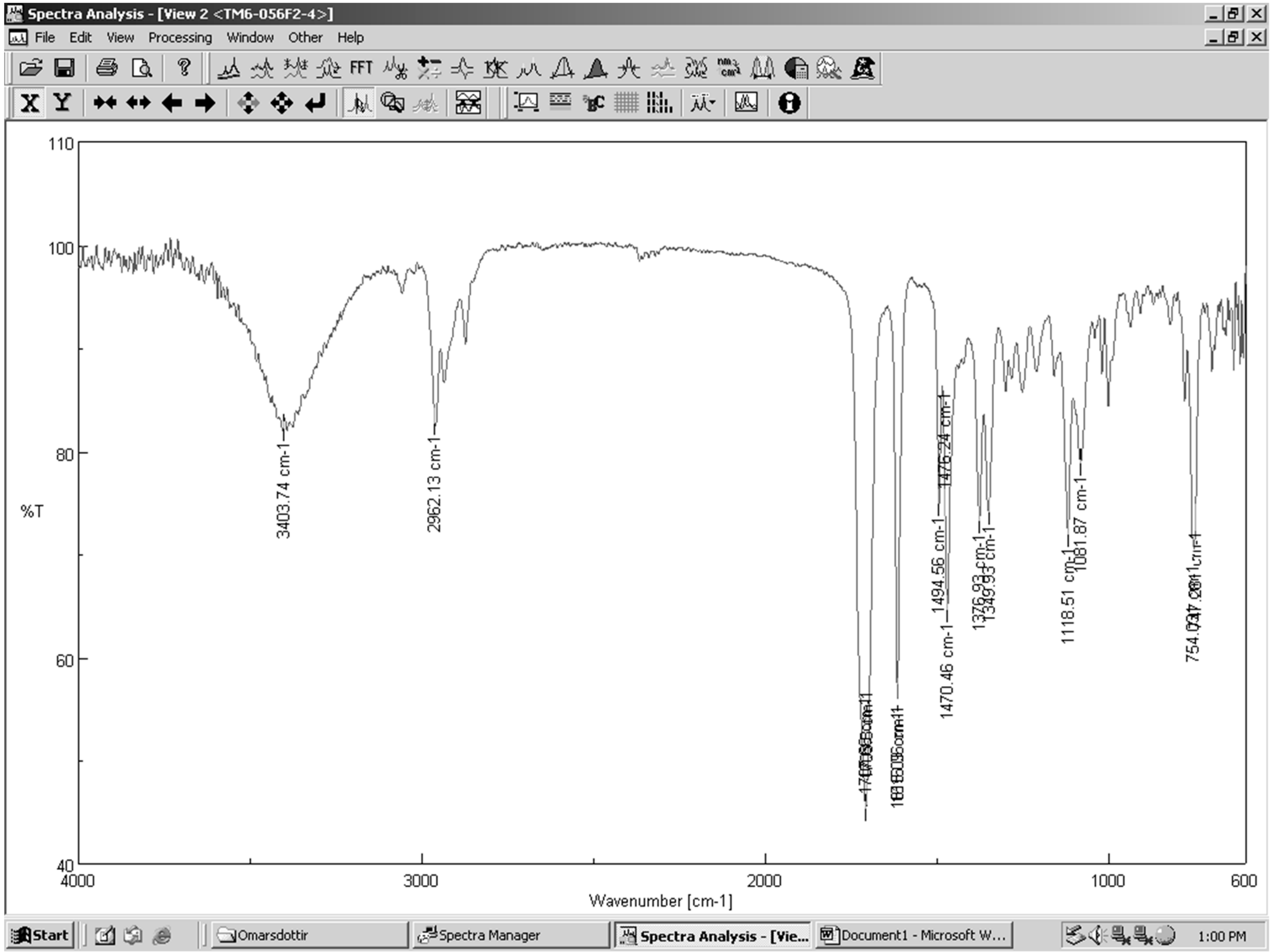

Figure S30. HRESIMS spectrum of (*R*)-3-propyldioxindole (**8**)

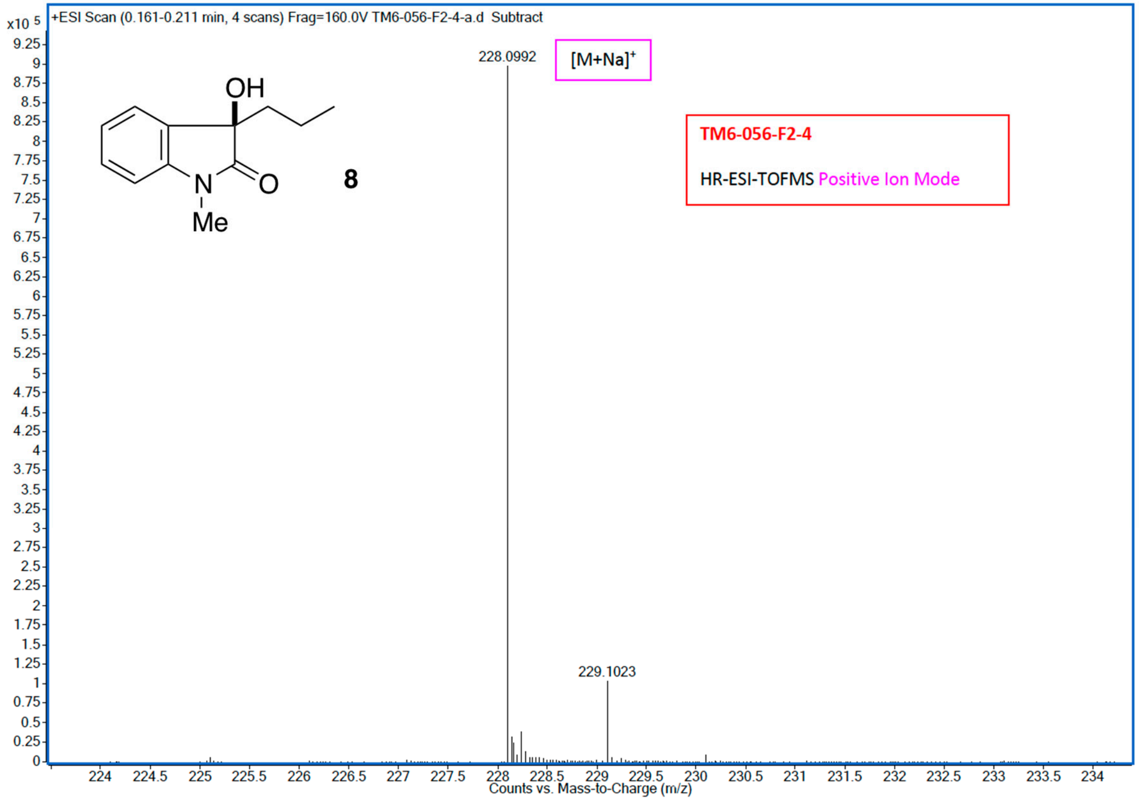

Search Results: Sample TM6-056-F2-4

Mass Measured	Theo. Mass	Delta (ppm)	Composition
228.0992	228.0995	-1.3	$[C_{12}H_{15}NO_2Na]^+$

Figure S31. UV spectrum of debromodihydrogeobarrettin A (**1a**)

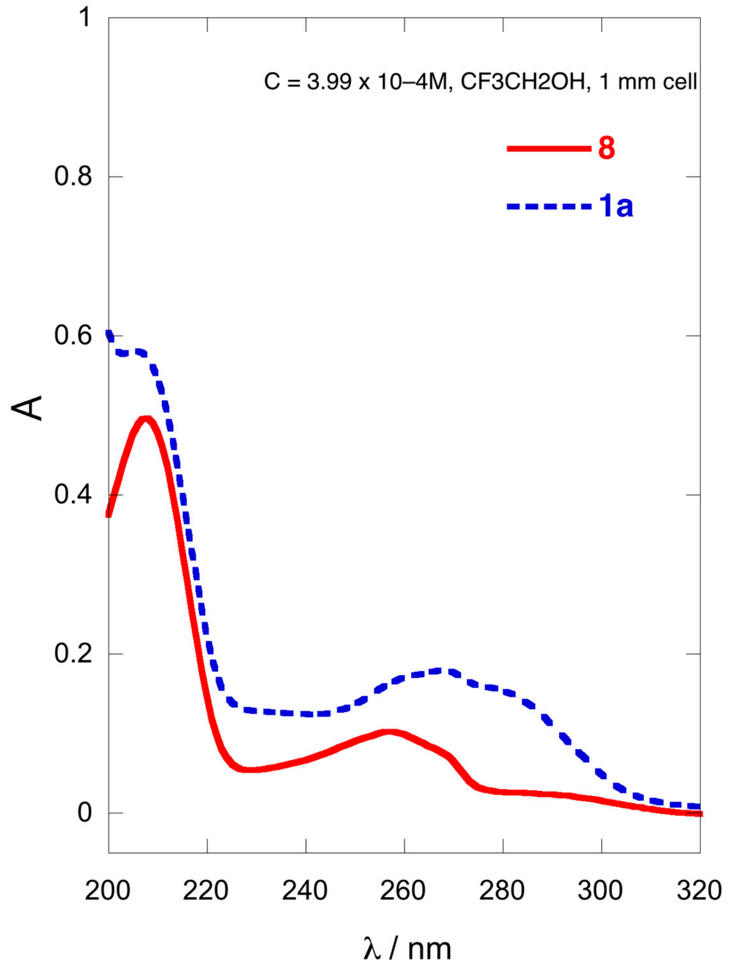

Figure S32. RP HPLC (C18) Chromatogram of L-DPT derivative of hydrolysate of debromodihydrogeobarrettin A (**1a**)

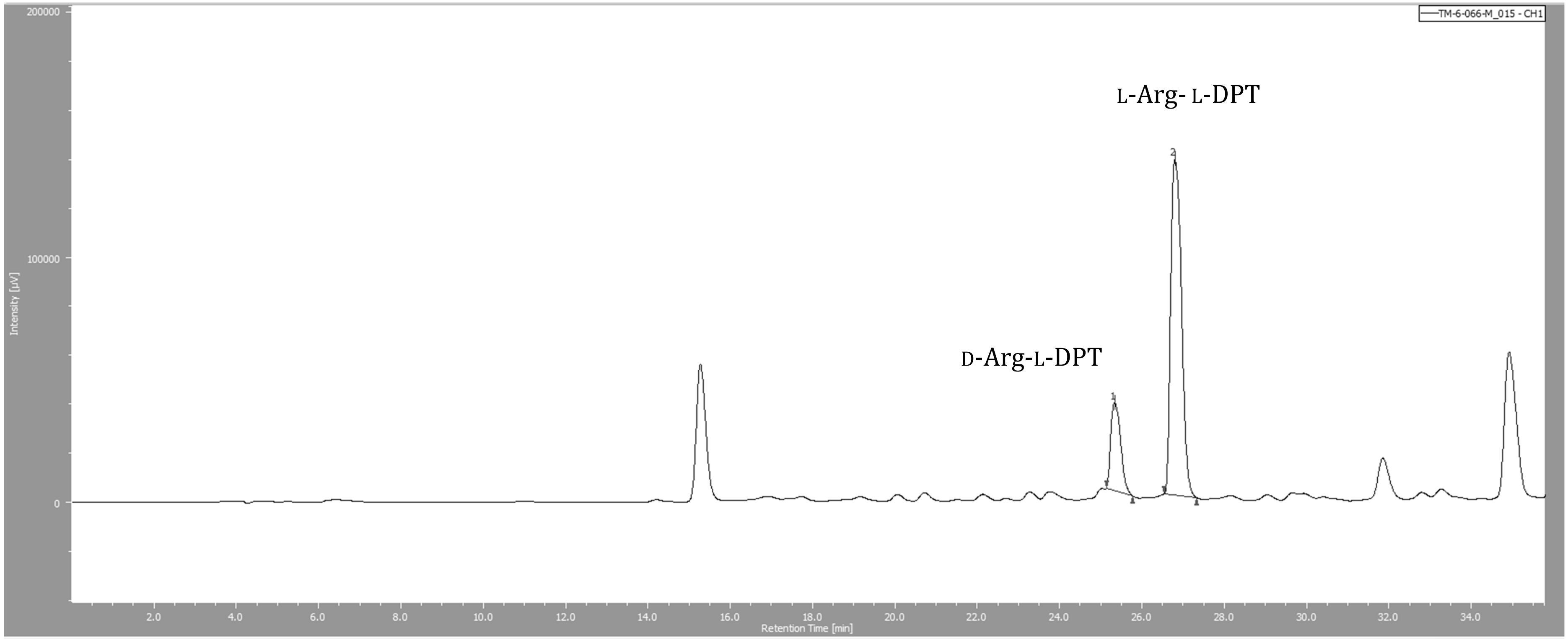

Peak Info. Table: TM-6-066-M_015

#	Peak Name	CH	tR	Area	Height	Area%	Height%	Quantity	NTP	Resolution	Symmetry Factor	Warning
1	Unknown	1	25.325	599585	35730	19.388	20.661	N/A	48110	3.038	1.396	
2	Unknown	1	26.783	2492985	137210	80.612	79.339	N/A	45790	N/A	1.386	
